# Identifying Biological Network Structure, Predicting Network Behavior, and Classifying Network State With High Dimensional Model Representation (HDMR)

**DOI:** 10.1371/journal.pone.0037664

**Published:** 2012-06-18

**Authors:** Miles A. Miller, Xiao-Jiang Feng, Genyuan Li, Herschel A. Rabitz

**Affiliations:** 1 Department of Chemistry, Princeton University, Princeton, New Jersey, United States of America; 2 Department of Biological Engineering, Massachusetts Institute of Technology, Cambridge, Massachusetts, United States of America; Michigan State University, United States of America

## Abstract

This work presents an adapted Random Sampling - High Dimensional Model Representation (RS-HDMR) algorithm for synergistically addressing three key problems in network biology: (1) identifying the structure of biological networks from multivariate data, (2) predicting network response under previously unsampled conditions, and (3) inferring experimental perturbations based on the observed network state. RS-HDMR is a multivariate regression method that decomposes network interactions into a hierarchy of non-linear component functions. Sensitivity analysis based on these functions provides a clear physical and statistical interpretation of the underlying network structure. The advantages of RS-HDMR include efficient extraction of nonlinear and cooperative network relationships without resorting to discretization, prediction of network behavior without mechanistic modeling, robustness to data noise, and favorable scalability of the sampling requirement with respect to network size. As a proof-of-principle study, RS-HDMR was applied to experimental data measuring the single-cell response of a protein-protein signaling network to various experimental perturbations. A comparison to network structure identified in the literature and through other inference methods, including Bayesian and mutual-information based algorithms, suggests that RS-HDMR can successfully reveal a network structure with a low false positive rate while still capturing non-linear and cooperative interactions. RS-HDMR identified several higher-order network interactions that correspond to known feedback regulations among multiple network species and that were unidentified by other network inference methods. Furthermore, RS-HDMR has a better ability to predict network response under unsampled conditions in this application than the best statistical inference algorithm presented in the recent DREAM3 signaling-prediction competition. RS-HDMR can discern and predict differences in network state that arise from sources ranging from intrinsic cell-cell variability to altered experimental conditions, such as when drug perturbations are introduced. This ability ultimately allows RS-HDMR to accurately classify the experimental conditions of a given sample based on its observed network state.

## Introduction

The development of high-throughput and multiplexed biological measurement techniques has led to the growing richness of data sets that describe biological networks [1]–[6]. These methods include particle-based and multiplex flow cytometric assays [7]–[9], kinase and protease activity assays [10], [11], and higher-throughput mass-spectrometry [2], [12], [13]. Such techniques not only allow for the simultaneous observation of multiple (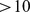) network nodes, but are of high enough resolution to capture complex nonlinear, high-order network interactions characteristic of many biological systems. When paired with systematic perturbation experiments, these methods can be used to infer network structure and understand the regulatory interactions among the network components. To achieve these objectives, a key challenge is to devise appropriate analysis tools that can handle the rich data efficiently and reliably.

Several network inference techniques have previously been developed for analyzing multivariate biological data. Network identification algorithms based on linearized steady-state models and regression analysis [14]–[18] are particularly effective in conditions of sparse sampling and noisy data. However, they often discount nonlinear interactions which may become significant in complex biological networks. To capture both linear and nonlinear interactions, Bayesian networks (BNs) [19], [20], clustering algorithms [21], [22], and information-theoretic approaches [5], [23], [24] have been employed. In some cases, BNs can infer directionality, causality, and allow for quantitative predictions of biological network responses [25]. Nevertheless, this capability can be limited by the high data-sampling requirements of the algorithm. Several nonlinear regression methods have an ability to predict biological network structures and their corresponding responses from multivariate and time-dependent data [26]–[28], although in general these methods do not readily support network structure inference while also efficiently allowing for the determination of higher-order cooperative statistical relationships.

This article introduces an adapted Random Sampling - High Dimensional Model Representation (RS-HDMR) algorithm for a nonlinear, deterministic, and predictive characterization of interactions among biological network components and their response to exogenous perturbations [29], [30]. RS-HDMR has previously been applied to a wide range of scientific (including biological) problems [31]–[34], and this work extends it to suit noisy, highly correlated data in biological network applications. From a multivariate data set, RS-HDMR extracts a hierarchy of low-order input-output (IO) relationships (termed RS-HDMR component functions) among the network components. These component functions are inherently nonlinear and have clear physical interpretations: they describe the independent and cooperative effects of perturbing one or more network components on the activity of other network components. Consequently, analysis of the RS-HDMR functions provides a quantitative understanding of the network interactions. In addition, the collection of these functions can serve as a fully equivalent operational model (FEOM) to predict the network response under previously unsampled conditions, including external perturbations. In this article, we further show that the network structure (that is, a map of the functional connections among the network components) can be generated from a global sensitivity analysis based on the extracted component functions.

As a general identification and interpolation technique, RS-HDMR has various advantages in bio-applications [29]. The operation of RS-HDMR does not require any mechanistic knowledge of the target network; the algorithm can perform even in the presence of unknown/unmeasured network components. Second, RS-HDMR analysis is robust against issues of over-fitting, sampling sparsity, and data noise. Third, RS-HDMR identifies the nonlinear and cooperative interactions, which can be important for biological networks, using an efficient and readily interpretable statistical framework. The inherent nonlinearity of RS-HDMR also enables the laboratory perturbations to go beyond the linear regime around the nominal state, which is a limitation of linear based methods. The data-sampling requirements of RS-HDMR scale favorably with the number of network nodes, therefore a moderate sampling effort is usually sufficient even with very large networks [35]. One result of this feature is that data discretization is generally not necessary for RS-HDMR analysis, hence information loss is minimized. In addition to inferring network structure, RS-HDMR can predict unsampled network response with in some cases better accuracy than several statistical methods that focus on prediction while not providing a clear interpretation of the underlying network structure. Lastly, RS-HDMR can be used to classify a network based on its observed state, again with accuracy in some cases better than that achieved by statistical techniques not associated with network structure inference. All of these properties render RS-HDMR an attractive technique for applications in systems biology and bioengineering.

Network species, or nodes, described by RS-HDMR can involve a wide range of biological entities, including proteins, RNAs, metabolites, and their combinations. In this proof-of-principle study, RS-HDMR was applied to several sets of cell-signaling data, including those used for benchmarking methods from the “Dialogue on Reverse Engineering Assessment and Methods” (DREAM) competitions [36]–[39]. This work chiefly focuses, however, on an application to a human T-cell signaling transduction cascade. Experimentally, single-cell intracellular protein expression and phosphorylation levels of the network nodes were simultaneously measured through multi-color flow cytometry, and the laboratory data was collected under nine different perturbative conditions [25]. RS-HDMR was implemented to analyze the laboratory data, resulting in a nonlinear quantitative input-output (IO) model of the network. The model can be utilized in both forward and reverse directions: it predicts the network response under previously unsampled conditions and it allows for the response of exogenous perturbations to be inferred by the observed network state. A map of network structure was also deduced and compared to network structures obtained through mutual information network analysis, through descriptions of the network in the literature, and through a previous Bayesian network analysis [25]. RS-HDMR was successful in identifying, with high-confidence, all but three of the first-order connections (connections between two protein species) well documented in the literature. The significant second-order RS-HDMR functions were shown to characterize several known feedback and cooperative mechanisms, which were unidentified through other methods in the T-cell signal transduction cascade.

## Results

### The RS-HDMR Algorithm

RS-HDMR is a tool to deduce nonlinear and cooperative interactions between a set of inputs and an output. In application to biological systems, input-output relationships include both direct biochemical reactions, such as protein-protein phosphorylation, and indirect interactions such as transcriptional regulatory events. The independent and cooperative effects of multiple input variables 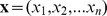 on an output 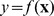 can be described in terms of a hierarchy of RS-HDMR component functions [29]

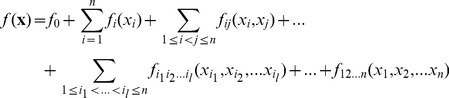
(1)Here 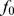 represents the mean value of 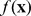 over the input sample space, the first-order component function 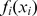 describes the generally nonlinear independent contribution of the input variable 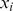 to the output, the second-order component function 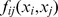 describes the pairwise cooperative contribution of 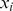 and 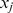, and further terms describe higher order cooperative contributions. In this application, we limit component functions to the zeroth, first, second, and third order. When the exact form of 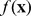 is unknown, the RS-HDMR component functions can be approximated by empirical functions, such as polynomials or splines, as long as they satisfy several orthogonality conditions. A more detailed description of the RS-HDMR algorithm can be found in the *Methods* and Supplementary Text S1.

Problems of over-fitting frequently arise when analyzing noisy, sparsely sampled, highly correlated data. We adapt RS-HDMR to these problems by employing a form of “model reduction”, where only inputs and their respective component functions measured to be significant at 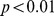 using the *F*-test are included in the RS-HDMR expansions [40] (Supplementary Text S1). Although not central to our aims here, precise type-I error quantification requires multiple hypothesis testing correction. RS-HDMR may alternatively incorporate other methods of regularization to deal with over-fitting, such as LASSO [41] or the Bayesian Information Criterion [42], which introduce penalty terms for the number of parameters in the model. Controlling false inclusions is especially relevant in application to network inference, where we aim to eliminate connections that proceed through measured intermediate nodes. We implemented a synthetic network model to demonstrate how variable selection can successfully address such issues (Supplementary Text S1, Table S1, and Figures S1, S2, S3, S4). Once the variables are selected, the coefficients describing each of the RS-HDMR component functions are calculated through Monte-Carlo integration and weighted least squares fitting (see *Methods*). The resultant expansion in Eq. (1) can then serve as a FEOM for predicting the network’s input-output relationships.

### Network Structure Identification by RS-HDMR

To characterize the network structure, the relative strength of network interactions is determined through a global sensitivity analysis based on the respective RS-HDMR component functions. In many applications of network structure identification, the measured nodes may not be defined *a priori* as being either inputs (strictly upstream) or outputs (strictly downstream) relative to other measured nodes in the network. In other words, causal relationships may not be defined *a priori* among network nodes. This is particularly the case when examining systems with inherent cyclical feedback mechanisms amongst the measured variables [43]. Time-dependent data can often be used to resolve directionality and causality within feedback mechanisms, and RS-HDMR can easily be applied to such data. Without time-series data, however, the mechanisms of interaction may not be strictly uni-directional. Reversible biochemical reactions can be driven in one direction or another and biochemical perturbations may have off-target effects. When measured nodes are not defined as strictly inputs or outputs, a separate RS-HDMR IO expansion can be formulated using each measured node as an output 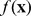 that is a function of the remaining network nodes. Consequently, 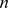 RS-HDMR IO mappings are determined for a system of 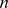 network components. For each of the 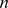 nodes, a single RS-HDMR model is trained for all experimental conditions, thus yielding a single, fully equivalent operational model (FEOM) of system behavior describing that node. The agglomeration of the 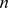 RS-HDMR expansions then constitutes a complete predictive model of network behavior with clear statistical and physical inference, where higher sensitivity indices correspond to significant connections that are more likely to be direct interactions (see *Methods*). For each RS-HDMR expansion, the total sensitivity/variance 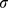 of the output 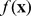 is decomposed into hierarchical contributions (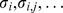) from the individual RS-HDMR component functions of the remaining input variables
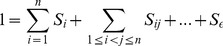
(2)In Eq. (2), 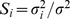 is defined as the sensitivity index of the corresponding RS-HDMR component function, 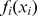. 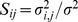 is the sensitivity index of the corresponding second-order component function, 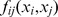. 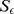 is the sensitivity index of the residual variation of the model (see Methods). The collection of sensitivity indices 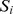, 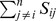, 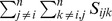 corresponding to first, second, and third order component functions of the input variable 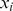 can then be summed into an index 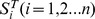, describing both independent and higher-order effects of 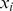 on an output. The magnitudes of 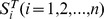 can be used to quantify the relative interaction strength between the outputs and the inputs.

For ready interpretation and visualization, network interactions described by a sensitivity index 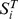 falling above a defined threshold value 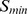 are considered significant/direct and are included in the map of the network structure. An insignificant network connection is defined as a biochemical interaction that likely proceeds indirectly through other measured network nodes. Several approaches have been used to define the optimal 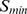, ranging from imposing an upper limit on the number of network connections allowed [20], to network structure averaging [44] and the Bayesian Information Criterion [45], [46]. In this work, 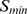 was defined empirically as 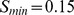 (see *RS-HDMR Identification of the T-Cell Signaling Network*). Network structure defined by lower and higher 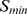 values are included in Supplementary Figures S5 and S6. The advantages of RS-HDMR in biological applications are summarized in the *Introduction* and will be demonstrated in the following sections.

### Single-Cell Data Analysis

The data used in this work are taken from high-dimensional cytometry measurements [25] where individual cells observed in a given population describe network behavior under statistically sampled microenvironments. Flow cytometry was used to simultaneously measure eleven different phospholipid and phosphorylated protein levels in individual cells 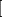
Akt (S473), Jnk, Raf, mitogen-activated protein kinases (MAPKs) Erk1 and Erk2, p38 MAPK, Mek1 and Mek2, protein kinase A (PKA) substrate phosphorylation, phospholipase C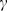 (PLC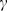), protein kinase C (PKC), phosphatidylinositol 4,5-bisphosphate (PIP2), and phosphatidylinositol 3,4,5-triphosphate (PIP3)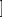. Nine data sets, each describing the same cell-signaling network but under different perturbative experimental conditions (Supplementary Table S2), were first analyzed individually using RS-HDMR. Eleven RS-HDMR IO mappings were determined from each data set to identify all significant connections among the eleven signaling nodes observed. Each IO mapping considered a single measured node as the dependent variable (the output, 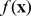) and the remaining ten nodes as the input variables. Every individual RS-HDMR mapping (99 total for this application) then provided a quantitative description of the nonlinear relationships between the output variable and its respective inputs.

In the second step, results from experimental conditions employing activation or inhibition of specific protein species (data sets 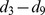) were paired with data taken from general stimulatory conditions (the control, 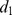 and 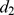) in order to examine the population-wide effects of exogenous perturbative (i.e., drugged) conditions. Specific perturbations were not directly observed through cytometry. Consequently, the measured levels of the perturbed node were discretized as either *high* (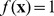) or *low* (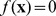) according to the relative exogenous perturbation, creating a “pairwise-comparison” dataset with a Boolean output. We define activating drugs as making their targets *high*, and inhibiting drugs as making their targets *low*. For example, when pairing the control data set 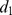 with data observed under PKA activating conditions (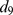), all PKA values in 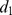 were uniformly set to low (0), and PKA values from 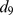 were set to high (1). RS-HDMR was applied to determine the effect of the specific activating or inhibitory cue on measured protein species, using the perturbed species (PKA in this example) as the output 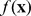.

### RS-HDMR Identification of the T-Cell Signaling Network

**Figure 1:**
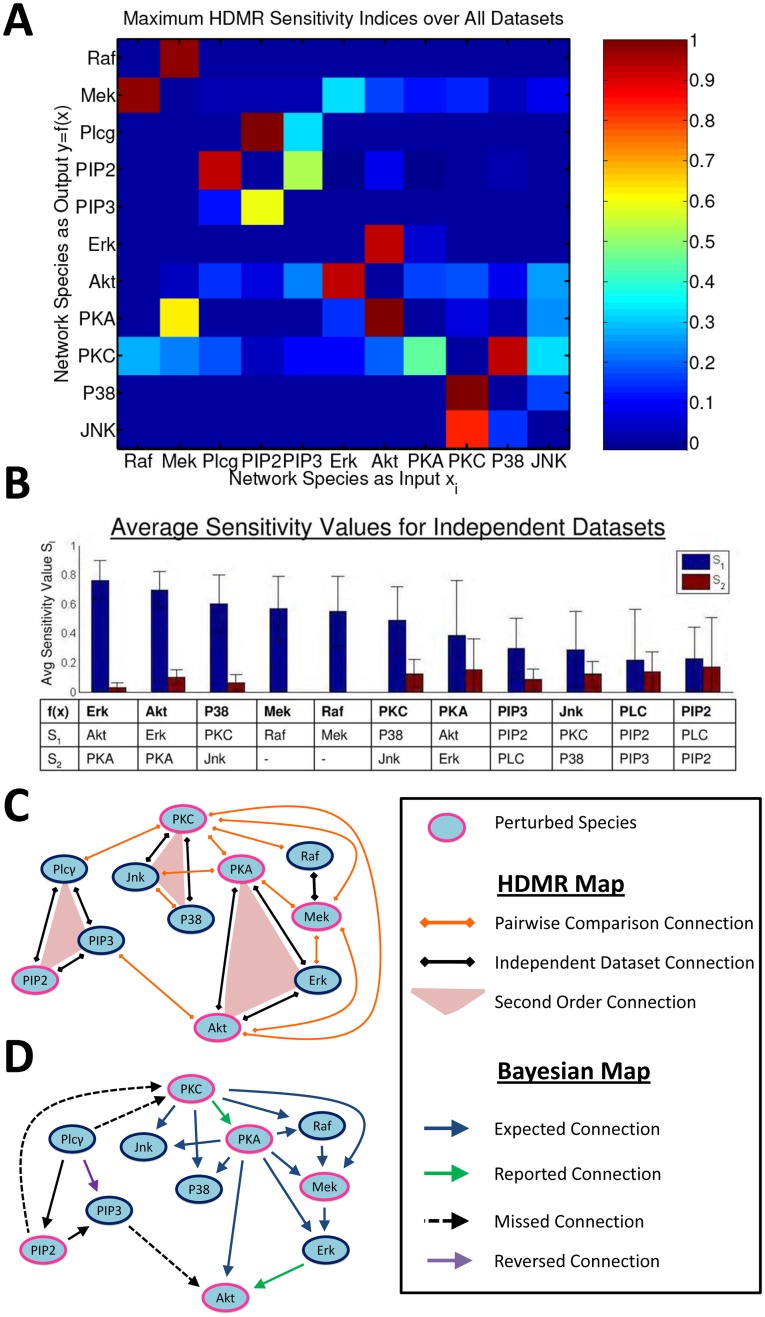
Network Inference Using RS-HDMR. (A) *Heat Map of RS-HDMR sensitivity indices.* The indices 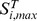 shown are the maximum 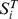 values among the nine individual data sets and thirteen pairwise comparison data sets. Network species on the ordinate describe the output 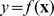, and species on the abscissa represent the inputs 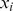. (B) *Sensitivity Indices 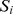 of First Order RS-HDMR Component Functions 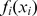.* First-order RS-HDMR component functions were calculated from all nine individual data sets, using each variable as the output 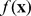. The first (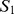) and second (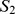) most significant functions were consistent across all nine data sets, and their average sensitivity index values 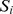 are reported. (C) *RS-HDMR Identified Significant Network Connections.* Significant network interactions (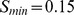) from individual and pairwise RS-HDMR analysis. (D) *Bayesian Network Analysis Identified Network Topology.* Reproduced from Sachs et al., 2005.

**Figure 2:**
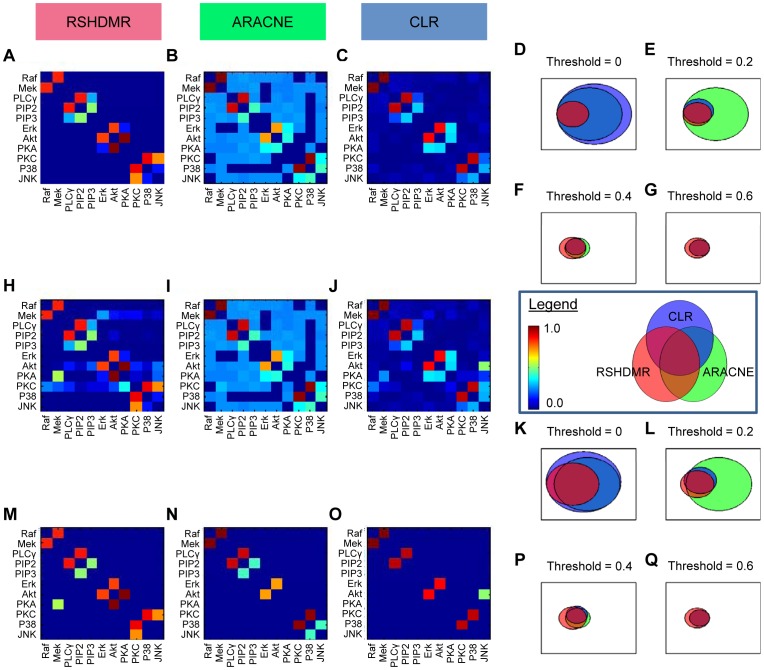
A Comparison of ARACNE, CLR, and RS-HDMR Network Inference. Network inference results both excluding (A–G) and including (H–Q) connections identified through pairwise comparison datasets. (M–O) Network connections with normalized edge weights 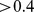. (A,H,M) RS-HDMR sensitivity indices, 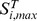. (H,M) Network species on the ordinate describe the output 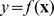, and species on the abscissa represent the inputs 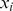, for the connections identified through pairwise-comparison. (B,I,N) ARACNE network inference results. (C,J,O) CLR network inference results. (D–G, K–Q) Venn diagrams comparing network connections identified with normalized edge weights above a defined threshold of 0, 0.2, 0.4, or 0.6. Circle areas scaled by the number of connections.

To generate an overall description of the eleven-node T-cell signaling network, sensitivity analysis results from the 99 RS-HDMR IO mappings utilizing individual data sets and the RS-HDMR IO mappings describing the thirteen (Supplementary Text S1) pairwise comparisons were aggregated. We calculated the maximum total sensitivity indices, 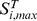, for each network connection under all experimental conditions (Fig. 1A). We compared the set of network connections defined by 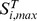 to connections previously described in the literature (Supplementary Table S3), to the most significant results from the BN analysis as presented in Sachs et al (Fig. 1D), and to mutual information based networks using the ARACNE and CLR algorithms (Fig. 2, see *Comparison with Mutual Information Methods*).

We identified twenty-one connections to be “high-confidence” using the threshold 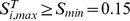, which corresponds to the lowest 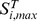 observed in the individual (rather than pairwise) data sets (Fig. 1C). Of these connections, all have been reported to some extent through previous experimental studies in a variety of systems (Supplementary Table S3). Three previously reported and well-known connections were ‘missed’ by RS-HDMR and only identified at a confidence below the threshold 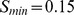 [PKA/Raf [47], PKA/p38 [48], and PKC/PIP2 [49], [50]]. RS-HDMR analysis successfully identified all but two of the connections revealed through the BN approach (Figs. 1C-D), as well as two additional connections well-established in the literature but not identified by BN analysis [PIP3/Akt [51], [52], PKC/Plc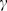 [49], [50], [53]]. Similar to BN analysis, RS-HDMR dismisses connections (or arcs) already explained by other identified arcs, thereby minimizing indirect relationships involving measured intermediate species. For example, Raf is known to activate Erk through an intermediate, Mek. RS-HDMR infers the interaction between Raf and Erk to be indirect. Moreover, RS-HDMR successfully identified indirect relationships defined through unmeasured nodes. For example, the RS-HDMR identified connection between PKC and p38 is known to proceed through unmeasured mitogen activated protein kinase kinase kinases (MAPKKK).

RS-HDMR analysis successfully identified several high-confidence second-order connections. Significant cooperative IO interaction generally occurred between nodes already described by significant first-order component functions. Three sets of three nodes each were observed to have significant second-order interaction among themselves: (1) PLC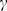, PIP2, and PIP3; (2) PKA, Akt, and Erk; and (3) PKC, Jnk, and p38. RS-HDMR analysis revealed the connections between PIP2, PIP3, and PLC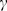 to be the most significant of the above three sets of second-order high-confident interactions. These three proteins are unique from other measured nodes in that they have significant negative feedback interaction. Activated PLC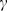 catalyzes the destructive cleavage of PIP2. PIP3, the product of PIP2 phosphorylation, serves as a docking site for PLC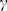 and ultimately catalyzes PLC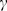 phosphorylation and activation. Evidence in the literature also supports the presence of complex feedback and cooperative interactions among Erk, Akt, and PKA [54]–[56]. Akt may interact with Erk through the Raf/Mek/Erk pathway and with PKA independently of Erk through a Calmodulin-dependent protein kinase kinase (CaMKK)-mediated pathway. However, PKA has been reported to negatively regulate Erk activity by phosphorylating Raf [57], [58]. In RS-HDMR expansions, these cooperative and/or feedback interactions are one explanation for the significant second-order RS-HDMR component functions observed.

### Comparison with Mutual Information Methods

ARACNE and CLR are two common mutual-information based network inference algorithms that, similar to RS-HDMR, are designed to (A) infer non-linear network connections that may/may not proceed through unmeasured intermediates, and (B) eliminate indirect connections that proceed through measured intermediates [5], [24], [59]. We applied these algorithms to the T-cell signaling data as a further comparison to RS-HDMR network inference. To implement ARACNE and CLR, the data from each of the nine individual data sets were discretized into 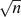 bins of equal frequency, where 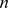 is the number of data points in a given set. We used an empirical estimator of mutual information and the most stringent threshold (0.0) for ARACNE’s Data Processing Inequality filter [59]. As with RS-HDMR analysis, we combined the maximum network connection scores over all of the individual data sets to generate the ensemble network structure. Figs. 2A-C juxtaposition the resultant network structures for RS-HDMR, ARACNE, and CLR, using data only from the individual data sets. The matrix symmetry reflects the non-directionality of the inferred networks, and network connections (“edge weights”) are normalized to have values between 0–1 for each graph. For the RS-HDMR case (Fig. 2A), the heat map values represent the maximum 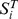 values observed regardless of which species was the HDMR output or input. Venn diagrams also compare the three methods: we use a threshold on normalized network edge weights to define a network connection as either present or absent (similar to 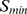 described above), and use Venn diagrams to depict the overlap in network structure for a given threshold (Figs. 2D-G). For this data, all RS-HDMR network edges are captured by both CLR and ARACNE at some non-zero value (Fig. 2D), suggesting RS-HDMR is not as sensitive as the other methods. However, the fact that RS-HDMR edges are a perfect subset of both the CLR and ARACNE networks suggests RS-HDMR has a low false-positive rate in detecting insignificant edges that proceed through measured intermediate nodes. At higher edge weight thresholds, the network structures become more consistent across the three inference methods, and the root-mean-squared difference (RMSD) between RS-HDMR and either of the other two networks is 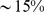. Figs. 2H-Q incorporate pairwise-comparison data sets into the network structures, where only network edges between the perturbed species (ordinate) and the other network nodes are added (consequently, the networks become asymmetrical). Venn diagrams compare these networks (Figs. 2K,L,P,Q), and Figs. 2M-O display only those network edges with a normalized weight of 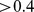 (corresponding to Fig. 2P, RMSD  = 0.17).

One of the most significant differences between RS-HDMR and the mutual information algorithms (with regard to network inference) is that RS-HDMR has the capability to infer higher order cooperative network interactions. This difference may explain some of the discrepancies between the algorithms’ results. For example, pairwise-comparisons have a relatively small impact on the number of network edges detected by either ARACNE or CLR; however, they dramatically increase the number of edges inferred by RS-HDMR. RS-HDMR identifies much more significant higher-order interactions in the pairwise-comparison data relative to inference within individual data sets, and these cooperative interactions heavily add to the network inference results. As another example, RS-HDMR tends to have more edges with high edge weights compared to CLR or ARACNE (Figs. 2M-O, P,Q). The edges that are strongest only in RS-HDMR inference (such as PKA–Akt and PKC–Jnk) tend to also have significant higher-order interactions with other nodes.

### Physical Interpretation of RS-HDMR Results

In addition to providing an overall description of network structure, RS-HDMR also serves as a tool for enabling a physical interpretation of network interactions. This is achieved by analysis of the individual RS-HDMR component functions and their relative contributions to the network behavior. For example, all of the nine RS-HDMR IO mappings formed with PKC as the output show the same two input variables to be most significant, quantified by the sensitivity indices of both their total and first-order RS-HDMR component functions (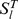 and 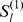, respectively). Each of the nine mappings describe PKC to be most sensitive to p38, with an average first-order sensitivity index (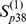) of 0.3. The second most significant component function in each RS-HDMR mapping corresponds with Jnk, having an average first-order sensitivity index (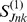) of 0.1 (Fig. 1B). The average RS-HDMR mapping with PKC as the output can be described with the following equations:
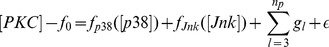
(3)

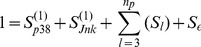
(4)where 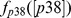 and 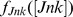 represent the first-order component functions of 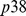 and 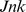, respectively, with corresponding sensitivity indices 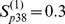 and 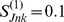. 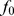 represents the zeroth-order component function, equal to the average response of 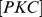. 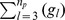 and 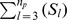 represent the remaining component functions in the expansion and corresponding sensitivity indices, respectively, where 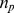 is the total number of significant component functions included in the model. 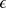 and 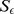 respectively describe the residual error of the model and its corresponding sensitivity index.

**Figure 3:**
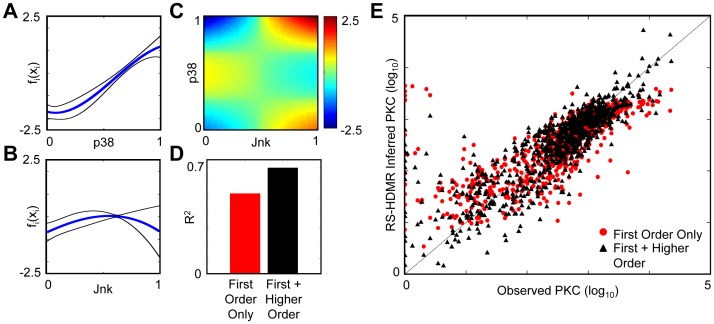
First and Second Order RS-HDMR Component Functions Describing PKC. (A-B) First-order RS-HDMR component functions describing interaction between inputs and an output, PKC, were averaged over corresponding RS-HDMR functions describing the same network connections under various experimental conditions. The thick line describes the mean function, and thin lines are one standard deviation above and below the mean function. (C) With PKC as the output variable 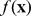, the heat map indicates 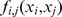 values as a function of 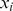 (p38) and 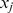 (Jnk) shown on the ordinate and abscissa, calculated from data set 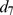. (D-E) The correlation coefficient 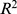 (D) and scatter plot (E) describe RS-HDMR fitting accuracy for predicting PKC in data set 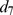, with or without including higher-order component functions. (A-C) p38 and Jnk are normalized to [0,1], and component function outputs are the same scale as (E).

As demonstrated by Fig. 3A–B, first-order component functions can show significant nonlinear behavior. Inspection of the shape of the component functions can provide meaningful physical insights. For example, the function dependence of PKC upon p38 is strongly positive, nearly linear, and consistent across several experimental conditions (Fig. 3A). In contrast, the function describing Jnk’s effect on PKC is much more nonlinear and only consistent under different experimental conditions at lower levels of Jnk. The function defining the relationship between Jnk and PKC is neither monotonic nor consistent across experimental conditions at high levels of Jnk, and consequently may be considered less significant (Fig. 3A).

Second and third order cooperative IO relationships were generally observed to be less significant than first-order dependencies in the T-cell signaling network. For example, all RS-HDMR expansions in data set 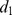 had total first-order sensitivity indices of 40% on average. The average total second and third order sensitivity indices were 5% and 2%, respectively. Nonetheless, several significant second-order terms were identified. Fig. 3C describes the second-order term with the highest sensitivity index (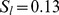) of the nine RS-HDMR expansions with PKC as the output. This term captures the cooperative influence of p38 and Jnk on PKC, as calculated from data set 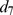. In this example, the cooperative influence is highest when both Jnk and p38 are high. Adding the significant higher-order component functions in this case increased the data-fitting quality of RS-HDMR by 40% (Fig. 3D-E) according to the correlation coefficient 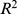.

Other significant cooperative interactions were identified among PIP2, PIP3, and PLC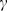, where second-order component functions accounted for up to 10% of the total observed variance. These identified higher order terms significantly improved data fitting and the predictive ability for several IO mappings. Inclusion of second order RS-HDMR component functions for data set 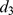, using PLC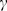 as the output 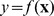, increased data-fitting quality so that the portion of RS-HDMR calculated data falling within 1% of observed values increased by 40%.

**Figure 4:**
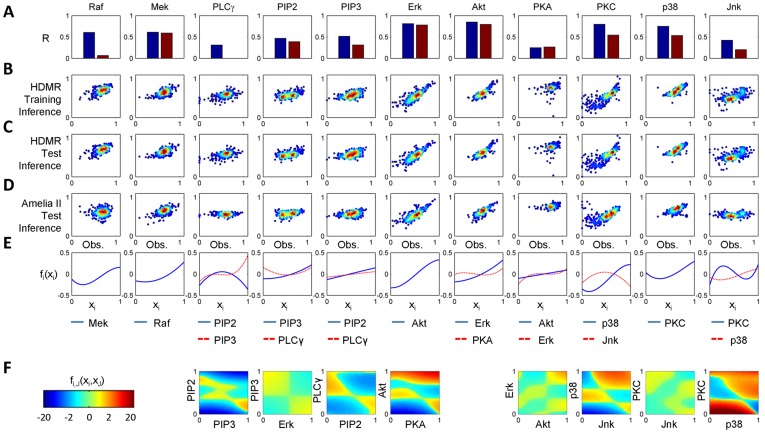
RS-HDMR Component Functions and the Predictive Capability of RS-HDMR Compared to Amelia II. RS-HDMR-generated FEOMs predict the values of network nodes (shown in columns) in a single cell based on the other node values in that cell, using data set 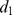. (A) Fitting accuracy described by the correlation coefficient, R, of the predicted vs. observed values of the test data for RS-HDMR (blue) and Amelia II (red). (B–D) Fitting accuracy scatter plots, where a higher density of data points is indicated by warmer color. Observed values are normalized to the maximum for each network node. (B,C) Observed vs. RS-HDMR inferred values of the training (B) and test (C) data. (D) Observed vs. Amelia-inferred values of the test data. (E–F) RS-HDMR component functions of the first (E) and second (F) order. Only the most significant second order function is shown, and the heat map indicates 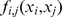 values as a function of 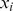 and 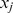 shown on the ordinate and abscissa. Inputs 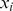 and 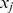 are linearly normalized to [0,1]. Component function outputs 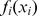 and 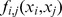 are normalized to the same scale as in B–D.

RS-HDMR second-order component functions are not constrained to pre-defined logic-based functions such as AND and OR gates, compared to some other methods [39]. However, in some cases RS-HDMR component functions are amenable to a logic-based interpretation. Fig. 4E-F shows the first and second order component functions corresponding to RS-HDMR expansions describing each of the eleven network nodes as the output in data set 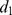. Several of the second-order component functions roughly follow the shape of an OR function, where 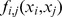 is high only when either 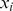 or 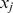 is high, but not both. This is particularly the case for the interactions between PIP2, PIP3, and PLC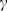. As another example, the second-order function relating the inputs p38 and PKC to the output Jnk resembles a NAND function, where the function is high only when both p38 and PKC are low.

### Network State Prediction

As described in previous works, the RS-HDMR functions can serve as a quantitative predictive FEOM when the explicit IO relationships among the network components are unknown. In the present application, RS-HDMR FEOMs can use incomplete information about the network state to predict unmeasured network properties. To illustrate, we use RS-HDMR to predict single-cell Akt levels based on observed values for the other network nodes in the same cell. 70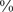 of the samples in data sets 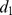 to 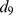 were randomly selected as the training set to identify the RS-HDMR component functions, which then served as an FEOM to predict Akt levels for the rest of the samples (the test set). Table 1 shows that for all data sets, 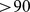
% of the Akt values predicted by the RS-HDMR FEOM fell within 20% of the laboratory values. We defined the sum of sensitivity indices 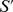 as the portion of total variance 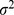 observed through first, second, and third-order interactions.
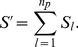
(5)


**Table 1:** Portion of Total Variance Accounted for by First-Order RS-HDMR Expansions for Akt and Relative Errors of First-Order RS-HDMR IO-mappings.

data set	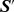 (Eq. 5)	1%	5%	10%	20%
*d*_1_	0.72	0.11	0.52	0.78	0.93
*d*_2_	0.85	0.16	0.60	0.80	0.96
*d*_3_	0.85	0.12	0.54	0.81	0.95
*d*_4_	0.93	0.28	0.88	0.97	0.99
*d*_5_	0.80	0.13	0.60	0.85	0.96
*d*_6_	0.47	0.07	0.33	0.65	0.93
*d*_7_	0.65	0.11	0.48	0.73	0.92
*d*_8_	0.90	0.18	0.68	0.85	0.97
*d*_9_	0.85	0.10	0.51	0.80	0.93

Accuracies are defined as the portion of data points falling within a given relative error range (1%, 5%, 10%, 20%) from the RS-HDMR-calculated value.

Table 1 indicates that 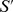 correlates qualitatively with the predictive accuracy of the FEOM. The residual variance 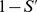 is due to higher-order (greater than third order) cooperative dependencies, measurement noise, and interaction with unobserved species. The ability of RS-HDMR to accurately infer the network response to an unsampled perturbation is a key advantage of the algorithm compared to other network inference algorithms. Fig. 4 compares the prediction ability of RS-HDMR to a multiple imputations algorithm, Amelia II, which was top-scoring in the DREAM 3 signaling-prediction challenge but is less applicable to network structure inference [37], [38], [60]. Briefly, Amelia II “fills in,” or imputes, incomplete data sets using an expectation-maximization algorithm with a bootstrap approach. To test the predictive capabilities of the two algorithms, 70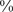 of the samples in the data set 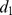 were randomly selected as the training set to identify both the Amelia II statistical model (see *Methods*) and the RS-HDMR component functions. The resultant FEOMs from both algorithms were then used to predict the test set values of individual network nodes for a single cell based on the measured state of other nodes in the same cell. RS-HDMR performs roughly as well as (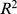 within 1%) or better than Amelia II in predicting the value of all eleven network nodes. In some cases RS-HDMR’s improved performance, such as in predicting PLCg, can be attributed to cooperative interactions where higher-order RS-HDMR component functions significantly add to the predictive accuracy.

**Table 2:** Dream3 Phosphoprotein Prediction Results.

Team	NSE	PVAL
Amelia II	3102	2 * 10^−22^
RS-HDMR No Noise	3250	3 * 10^−22^
Team 106	3310	4 * 10^−22^
RS-HDMR Noise	3500	6 * 10^−22^
Team 302	11329	7 * 10^−14^

Numbered teams are as of yet unnamed participants. Inference performance is judged by the normalized square error (NSE) and corresponding p-value (PVAL). “Noise” and “No Noise” refer to whether or not training data was pre-processed with multiplicative noise.

**Figure 5:**
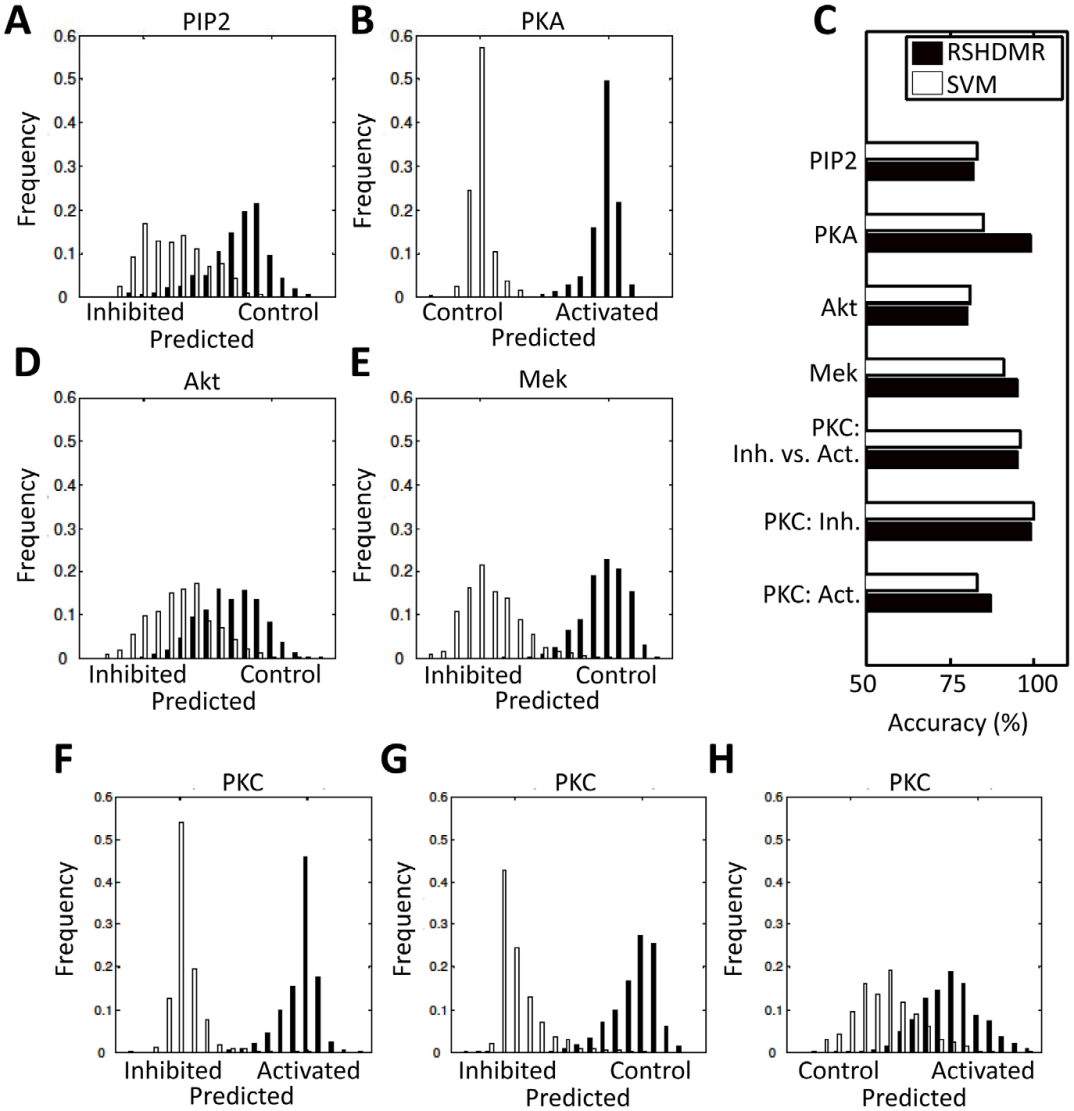
Inverse FEOMs Infer Experimental Conditions. Inverse FEOMs were constructed between data sets describing the network under general stimulatory (“Control”) and specifically perturbative (“Activated” or “Inhibited”) experimental conditions. The perturbed node was used as the output, whose values were digitized according to experimental conditions, being either relatively high (1) shown in black or low (0) shown in white. (A–B, D–H) These histograms describe RS-HDMR-fitting ability for data observed under activating or inhibiting conditions. Although the experimental perturbations are approximated as discrete, RS-HDMR expresses the model output as continuous, thus the distribution of RS-HDMR fitted results approximately resembles two Gaussian distributions. Clear separation of the two distributions for a given plot indicates good RS-HDMR prediction of the corresponding perturbation. (C) Inverse FEOM accuracy using a SVM classifier and RS-HDMR. RS-HDMR accuracy corresponds to histograms in A–B, D–H.

We also constructed HDMR FEOMs to infer experimental conditions based on the measured network state. For pairwise-comparison of two experimental conditions, datasets describing the signaling network under generally stimulating conditions were combined with datasets describing the system under specifically perturbative experimental conditions (see *Single-Cell Data Analysis*). The experimentally targeted nodes are defined as the output rather than input variables in this case; hence, we call these “inverse” FEOMs. In this particular application, the laboratory values for the outputs are Boolean (either inhibited (0) or activated (1)), while the corresponding RS-HDMR predictions generate continuous values, forming two Gaussian-like distributions (Fig. 5). When these two distributions are clearly separated, it indicates that the inverse FEOM can reliably distinguish/predict the two perturbation conditions. When the perturbations have higher resolutions (e.g., high, medium, and low perturbations), the inverse FEOM can similarly deliver more quantitative predictions. Nonetheless, the inference problem is a simple two-category classification when the perturbations are Boolean.

**Table 3:** Dream3 Cytokine Release Prediction Results.

Team	NSE	PVAL
Team 106	4460	8 * 10^−36^
RS-HDMR Noise	7330	2 * 10^−25^
RS-HDMR No Noise	11100	5 * 10^−15^
Team 302	14000	3 * 10^−09^
Team 126	29800	1 * 10^00^

Numbered teams are as of yet unnamed participants. Judging by the normalized square error (NSE) and corresponding p-value (PVAL), RS-HDMR performed second most accurately. “Noise” and “No Noise” refer to whether or not training data was pre-processed with multiplicative noise.

An analysis of the inverse FEOM data shows that perturbation of a node upstream of other measured variables in a signaling cascade affects the system more significantly. Consequently, RS-HDMR is able to accurately reveal the differences between the perturbed and generally stimulated networks. On the other hand, downstream nodes can be expected to not directly impact other measured nodes in the network when activated or inhibited. To define accuracy for the inverse FEOMs, we use a threshold of 0.5 to group RS-HDMR output as one of the two Boolean values (0 or 1), and calculate the portion of data that are correctly modeled as falling above or below the threshold based on their respective experimental conditions. Perturbations of PKA, a protein previously observed to be an upstream node in the T-cell signaling cascade [47], [48], [61], [62], are well mapped by RS-HDMR (Fig. 5B). RS-HDMR mapping determines from which conditions a given data point was observed in, with over 99% accuracy, when comparing data observed under PKA-activating conditions versus general stimulatory conditions. In contrast, perturbations of Akt, a species previously reported as downstream in the signal cascade [63]–[66], show much less effect on network behavior. Comparison between data from general stimulating and Akt-inhibited conditions yields a significantly lower RS-HDMR fitting accuracy of 80%. PIP2 also showed a lower RS-HDMR fitting accuracy of 82%. The detectable effect of perturbations on network behavior was observed to be significant for Mek and PKC perturbations, resulting in inverse FEOMs with 95% and 99% accuracy, respectively. Because PKC was inhibited and activated in two separate datasets, it was possible to make a comparison between each dataset observed under the perturbed condition and the control dataset, as well as directly between the two datasets observed under perturbative conditions. As evidenced in Fig. 5F-H, RS-HDMR best identifies differences in network behavior between the two datasets observed under specific perturbed conditions. We implemented a well known method, a support vector machine (SVM) classifier, to categorize the network states and benchmark RS-HDMR inference accuracy (Fig. 5C). In all cases, RS-HDMR performs roughly as well as (accuracy within 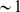
%) or better than SVM while having the advantage of also providing network structure inference.

### Robustness of RS-HDMR Results to Sample Size and Data Noise

The performance of many network identification algorithms, including BN analysis, are sensitive to the data sample size being analyzed. The data sample size used in the individual dataset RS-HDMR analyses was reduced to 25% of the original size to similarly test the sensitivity of RS-HDMR network identification capability. As with analysis of the full data sets, multiple subsets of data were generated and analyzed for consistency purposes. The effect of truncated sample size on the individual RS-HDMR expansions was different for each of the 99 RS-HDMR mappings. In several cases, reduced data size led to complete loss of calculated first and second order interactions determined to be significant by the *F*-test, resulting in a collapse of the RS-HDMR expansions to only the zeroth-order term (such that 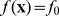). In most cases, however, first-order interactions were still captured, with insignificant effect on data-fitting and predictive accuracy as compared to mappings derived from the entire data set. In this application, network structure identification through RS-HDMR sensitivity analysis was robust to the tested decrease in sample size. All of the ten high-confidence first-order network connections indentified by using the full data sets were also captured through RS-HDMR analysis of the truncated data sets. Significant second and third order functions were still observed in many data sets, although higher-order mapping was slightly more sensitive to data truncation.

The robustness of the RS-HDMR analyses to noise in this application was tested through the addition of artificial noise beyond that naturally present in the experimental data. Noise was increased in the system by the addition of a random number 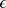 to the measured value 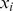, such that 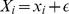. 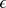 was chosen from the normal distribution N(0,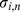), with 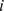 being a given node in data set 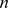, and 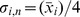. The effect of additive noise to individual RS-HDMR expansions varied such that while some mappings were insignificantly affected, several lost all significant first and second order component functions. This also occurred in the RS-HDMR analysis of the truncated data sets. Generally, both noise and reduced sample size mostly affect accurate identification of component functions previously described by lower sensitivity indices. Added noise or small sample size potentially masks weak network connections, leading them to be excluded from the RS-HDMR formulation. In this work, however, the aggregated RS-HDMR sensitivity results proved to be robust to the increased stochasticity. All ten of the high-confidence first-order connections identified through single data set analysis were captured using the noisy data. As with the effect of reduced sample size, the higher-order RS-HDMR analyses were more sensitive to the artificial noise than the first-order connections. Most of the second and third-order interactions identified using the original data set were observable with added noise, although some previously significant higher-order component functions lost significance.

### Benchmarking RS-HDMR Performance with the DREAM Challenges

The DREAM project organizes reverse-engineering challenges that are open to the research community [36], [37]. The past two challenges from Dream3 (2008) and Dream4 (2009), in the categories of “Signaling Response Prediction” and “Predictive Signaling Network Modeling,” respectively, are published online (http://wiki.c2b2.columbia.edu/dream). We have analyzed these challenges using RS-HDMR to further demonstrate the algorithm’s broad applicability and to compare its performance to other computational methods used in the field.

**Figure 6:**
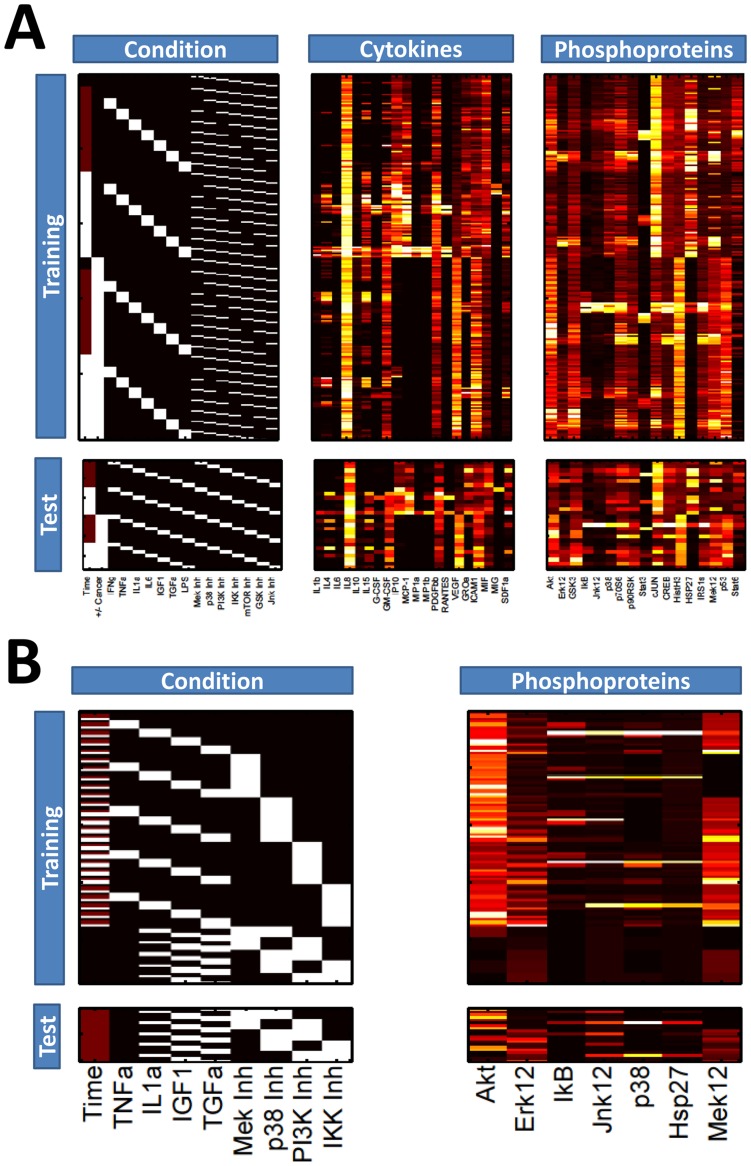
Dream Challenge Data. Data for the Dream3 (A) and Dream4 (B) challenges are presented as heat maps, where lighter color indicates higher value (generally concentration). For the “Condition” arrays, columns may represent whether a condition (e.g., a growth factor or inhibitor) is present (white) or absent (black). For Dream3 data, the “+/− Cancer” column describes whether the cell-type is normal (black) or cancer (white). Each row in either the “Phosphoproteins” or “Cytokines” array corresponds to the adjacent row in the “Condition” array. Arrays labeled “Training” were used to identify RS-HDMR component functions, which then served as FEOMs to predict network behavior in the “Test” arrays.

Dream3 and Dream4 challenges are similar in both the experimental data and the prediction task. Briefly, the challenges explore the extent to which cellular signals and behaviors can be predicted in response to various extracellular cytokines, growth factors, and signaling inhibitor drugs [67]. The Dream3 challenge provides a training set of data that describes the secretion of 20 cytokines and the signaling activities of 17 phophoproteins in response to a panel of inhibitors, growth factors, and cytokines, across three time points (Fig. 6A). Furthermore, two cell lines are analyzed: human normal and cancerous (HepG2) hepatocytes. Measurements of cytokine release and phosphoprotein activity were obtained using the Luminex xMAP sandwich assay, under a total of 122 conditions/time-points for each cell type. The challenge is to accurately predict the cytokine secretion and signaling activities in response to conditions that are not included in the training set. The Dream4 challenge is fairly similar, but explores fewer conditions/time-points (95), uses only one cell type (HepG2), measures only signaling activities of seven phosphoproteins, and applies fewer growth factors, cytokines, and inhibitors (Fig. 6B). Unlike Dream3, the Dream4 challenge provides a proposed network structure culled from the literature that is to be potentially incorporated into modeling efforts (Supplementary Figure S7A). RS-HDMR has the capability to incorporate prior information regarding network structure. However, for this work we tackle both Dream3 and Dream4 challenges using RS-HDMR to infer network structure and predict network behavior with no *a priori* information of the network structures.

**Figure 7:**
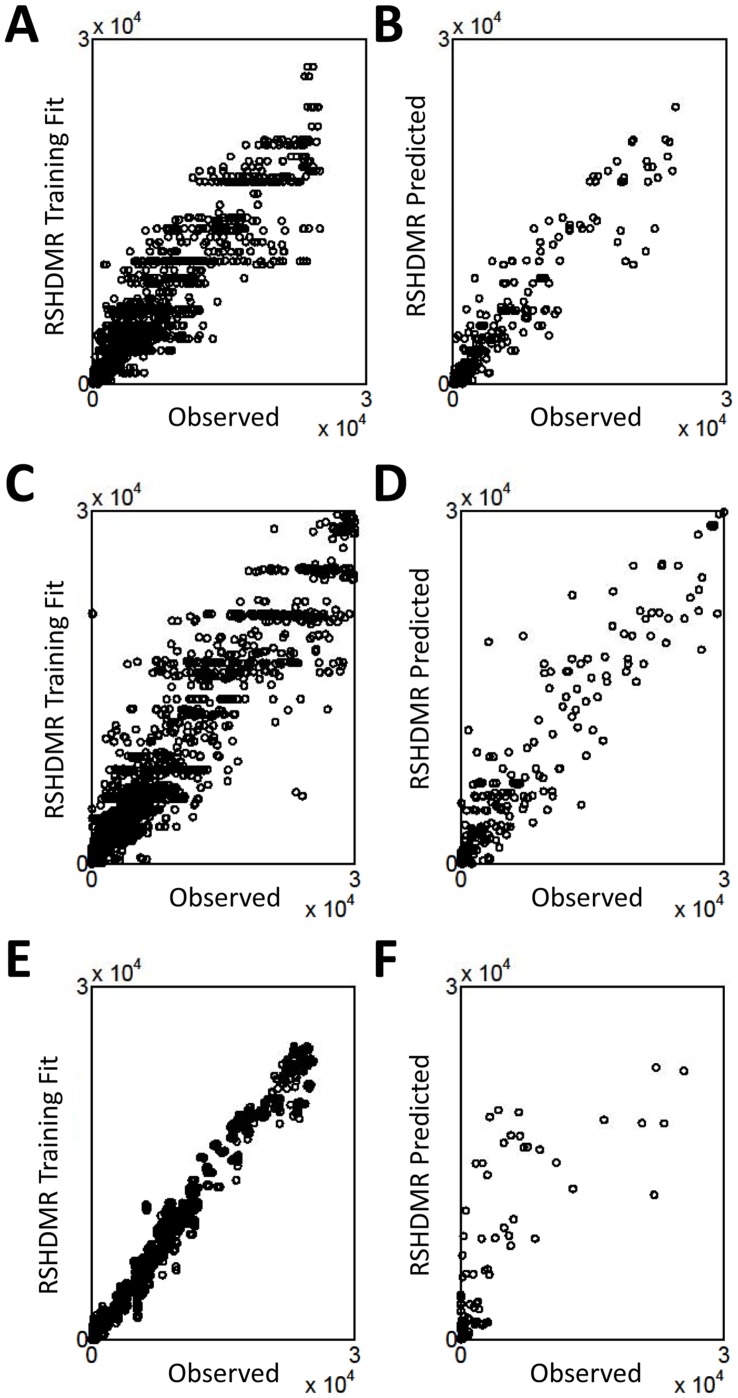
Dream Challenge Prediction Accuracy. Scatter plots describing the observed vs. RS-HDMR predicted values of the training (A,C,E) and test (B,D,F) data from the three Dream challenges. (A,B) Dream3 challenge phosphoprotein prediction. (C,D) Dream3 challenge cytokine release prediction. (E,F) Dream4 phosphoprotein prediction.

We implement RS-HDMR in a manner consistent with the DREAM competition guidelines: we organize the training and test data in a naive manner by defining experimental conditions (e.g., time, IL-1a stimulation, presence of inhibitor), as inputs used to predict an output (e.g., IL-1b secretion, phospho-Akt concentration). We model time explicitly by considering it as an input variable, such that RS-HDMR captures temporal network structure dependencies through higher-order RS-HDMR component functions between time and other input variables. Outputs are known in the training data and allow for us to infer an FEOM of system input-output relationships based on RS-HDMR component functions. We then use this FEOM to predict unknown outputs in the test data, based on the given experimental conditions for that data. Fig. 6 depicts each input variable as a column in the “Condition” array, and each output variable as a column in either the “Phosphoproteins” or “Cytokines” array. We identified RS-HDMR component functions describing the IO relationships within each training set and used these functions as FEOMs to predict cellular response to conditions in the test data. For the Dream3 challenge, we identified separate RS-HDMR component functions for each of the two cell-types (indicated as “+/− Cancer” in Fig. 6A), and consequently employed the two sets of component functions as cell-type specific FEOMs. The predictive accuracies of the RS-HDMR FEOMs for each challenge are described in Tables 2, 3, 4 and shown graphically in Fig. 7. For the Dream3 challenges, fitting accuracy was judged by normalized square error (NSE) and an associated p-value, described in detail elsewhere [37]. For the Dream4 challenges, the overall score was a function of both the sums of squared errors and the number of network edges used in the modeling [37]. We report a network structure consisting of 23 significant first-order RS-HDMR component functions with associated network edges (Supplementary Figure S7B), although many higher-order functions among these 23 interactions contributed to the predictive model.

The higher dimensionality and sparse sampling of the Dream data compared to the single-cell T-cell data make RS-HDMR inference relatively ill-conditioned, especially considering all input variables except “time” are sampled at only two concentrations over all experimental conditions. To address this issue, we implemented RS-HDMR after applying a small amount of Gaussian multiplicative noise (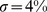) to the training data input variables that was representative of the expected experimental variation [37], [39]. We repeated this procedure ten times to average the stochastic effects and compared the results to RS-HDMR inference without added noise (Tables 3, 4). For the Dream3 challenge this procedure significantly improved RS-HDMR prediction of cytokine release and reduced the NSE by roughly 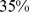. Individual RS-HDMR expansions are calculated for each output variable (i.e., each phosphoprotein or cytokine), and adding noise decreased the RS-HDMR computation time by an order of magnitude for several of the RS-HDMR expansion calculations because of faster solution conversion. For the Dream4 challenge, which had even fewer training data than Dream3, we added noise to the training data as above but also increased the sample size. We expanded each data point in the training set to ten data points with a centroid of the original data point and a gaussian standard deviation of 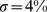, increasing the training data sample size from 95 to 950. This over-sampling procedure improved the algorithm’s “prediction score” by over 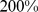. To mimic the original competition we optimized data pre-processing by cross-validating with a masked subset of the training data, before analyzing the test data.

RS-HDMR performs well in the Dream3 and Dream4 challenges in spite of the fact that the Dream challenge experimental designs and data do not fully exploit RS-HDMR’s advantages. For example, the experimental conditions tested (e.g., the concentrations of cytokines and inhibitors) are discrete rather than continuous. Furthermore, the data describes population averages rather than single-cell values, and is thus further from the ideal “random-sampling” experiments for which RS-HDMR is better suited.

## Discussion

BN analysis was previously employed to characterize the protein-signaling network from data used in this work [25]. BN analysis is similar to RS-HDMR in that it serves as a powerful tool to characterize network interactions from stochastically sampled multivariate data. However, BN analysis is functionally different from RS-HDMR in several respects. BN analysis is most advantageous in providing a framework for inferring causality through the analysis of probabilistic dependencies. Network connectivities can be defined within a BN framework frequently through a multinomial model using discretized variables or through a multiple regression model. Network connections described by linear regression models generally fail to effectively capture nonlinear interactions typical of biological systems [68]. To address this issue, nonparametric regression models have been employed, but often without efficient calculation of cooperative interactions [27]. Another common form of BN analysis is described by multinomial distributions of discrete nodes, which allows for the identification of both nonlinear and cooperative network interactions. However, the discretization process often leads to decreased inference resolution and information loss.

To address these issues, RS-HDMR was used in this work to quantitatively characterize network interactions through construction of continuously distributed high-dimensional IO functions. Nonlinear characterization of IO relationships is made computationally manageable without losing significant information by approximating interactions through a hierarchy described by orthonormal basis functions. The approximation of the RS-HDMR component functions with orthonormal basis functions still allows for a clear physical/statistical interpretation while maintaining relative robustness to outliers [69]. Recent developments of the HDMR algorithm further demonstrate how the method can robustly apply to various ill-posed problems, including systems with correlated variables, and variations of the method have been successfully applied to noisy and underdetermined systems as well [70]–[72]. The RS-HDMR technique should scale well with large numbers of species and with a modest amount of data, as each data point will generally project onto all of the variables to permit the identification of each individual contributing component function [70]. The basis functions used in HDMR do not impose any restriction on the types of biochemical interactions. In principle, any basis functions can be used to approximate the HDMR component functions. The advantage of orthonormal polynomials is that the solution is unique [73]. The imposition of an orthogonality requirement in the RS-HDMR expansion is consistent with the expectation that interacting network components are likely dominated by low order terms. The orthogonality feature also permits the determination of selected terms of fourth-order and greater order as warranted, for example to describe protein complexes of greater than three directly interacting components. Although we utilize polynomial basis functions in this work, alternative functional forms could be implemented within the RS-HDMR framework to better capture particular biological interactions, such as saturable processes. Similar to many network inference methods, RS-HDMR cannot explicitly capture feedback relationships from data collected at a single time-point. However, the basic HDMR framework could readily be applied to identify feedback relationships from suitable time-series data. Ultimately, experimental data collection, especially in the context of cell-signaling networks, is generally the greatest limitation in capturing statistically significant higher-order and non-linear interactions when few *a priori* assumptions or constraints are made regarding network structure. RS-HDMR has the advantage of being highly efficient in extracting these interactions from sparse and noisy experimental data [35], [74].

**Table 4:** Dream4 Signaling Prediction Results.

Team	Overall	Edge	Pred.	Akt	Erk1/2	IKB	Jnk12	p38	HSP27	Mek12
	Score	Num.	Score	PVAL	PVAL	PVAL	PVAL	PVAL	PVAL	PVAL
Team 441	6.678	18	8.167	−4.3	−8.8	−9.4	−9.8	−8.0	−10.1	−6.8
Team 476	6.324	17	7.73	−3.0	−13.5	−8.2	−9.9	−9.9	−5.3	−4.4
Team 533	6.279	26	8.43	−4.7	−15.7	−9.4	−9.4	−5.7	−5.7	−8.3
RSHDMR	5.67	23	7.56	−4.4	−9.6	−7.6	−9.2	−4.0	−10.9	−7.3
Team 491	5.016	18	6.505	−3.1	−8.0	−7.9	−10.3	−4.3	−6.0	−6.0
Team 451	4.688	17	6.094	−4.5	−10.2	−7.0	−3.6	−5.0	−7.9	−4.4
Team 256	4.58	22	6.4	−4.2	−7.6	−9.0	−10.1	−3.8	−3.6	−6.6
Team 395	3.719	15	4.96	−5.6	−9.1	−3.6	−4.3	−1.6	−2.2	−8.3
Team 314	3.097	27	5.33	−3.8	−4.8	−6.6	−9.2	−3.9	−3.4	−5.8
Team 544	2.209	18	3.698	−3.0	−6.7	−4.4	−3.9	−1.7	−3.7	−2.5
Team 504	1.545	10	2.372	−2.9	−4.7	−1.8	−2.9	−2.3	−1.3	−0.6
Team 347	0.403	19	1.974	−3.2	−0.1	−4.0	0.0	−2.6	−2.9	−1.1
Team 471	0	54	4.467	−3.0	−7.2	−3.4	−10.4	−2.9	−2.4	−1.9

Numbered teams are as of yet unnamed participants. RS-HDMR inference was the fourth most accurate, as measured by the “prediction score,” which is an overall metric that incorporates p-values (PVAL) describing the statistical significance of prediction for each of the phosphoproteins. P-values are log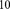 transformed.

In terms of network structure inference capability, RS-HDMR results are fairly similar to those obtained through other methods tested here, including BN analysis, ARACNE, and CLR. RS-HDMR captures roughly 90% of the connections identified by BN analysis, while capturing several additional network connections that have been discussed in previous literature. Likewise, RS-HDMR largely identifies the same networks connections as ARACNE and CLR (RMSD 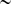
15% for such comparisons). Results suggest that inference differences between RS-HDMR and the other methods tested can be explained in part by cooperative non-linear interactions that RS-HDMR alone captures, such as the well-documented relationship among PLC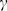, PIP2, and PIP3. Network inference accuracy can depend on the underlying structure of the network, and benchmarking signaling network inference has been challenging in part due to a lack of good gold-standard metrics in the field [37], [75]. In this work, we use the accuracy of RS-HDMR with regards to network response prediction and classification as one metric of network inference accuracy.

As a predictive model of network behavior, RS-HDMR FEOMs can use partial information about network state to infer unknown network properties. This feature has applicability ranging from controlling network behavior to optimal experimental design. For example, the predictive properties of RS-HDMR may be especially useful when facing constraints on the number of network nodes one can reasonably measure in an experiment. Furthermore, we demonstrate that RS-HDMR inverse FEOMs can discern key differences in network behavior that arise from a variety of exogenous perturbations. The concept of using the network state to infer experimental conditions can be extended to a wide array of biological applications. For example, biological network state has been used in previous studies for the prediction of cellular phenotypes ranging from embryonic cell fate decisions to epithelial cell migration speeds [20], [76].

More than a tool to map causal network interactions, RS-HDMR serves as an algorithm to develop a quantitative FEOM of network interactions which capturing direct, indirect, and cooperative nonlinear interactions. The algorithm is well-suited to capturing the effects of exogenous perturbations on network behavior and can incorporate this information into the overall network structure, as demonstrated in the T-cell signaling network application. The hierarchical framework of RS-HDMR supports the facile incorporation of priors that are usually hierarchical by nature. For example, RS-HDMR variable selection procedures can be modified to define *a priori* which component functions are included in the network structure. More detailed information may also be incorporated beyond whether or not the network connection exists. For example, the weight and/or functional form of the first-order component function describing the relationship between two proteins may be defined *a priori* from previous binding studies. This prior component function could then be subtracted from the output 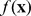 before computing the rest of the RS-HDMR expansion. Likewise, higher-order priors, which could for example arise from information about protein complexes, may also be incorporated in a similar manner. Finally, prior information regarding causality (e.g., as is present with time-series data) can be incorporated either in the variable selection process or after the RS-HDMR expansion has been solved, at which point component functions are merely labeled as directional.

Higher-order interactions can be a significant factor in the characterization of network topology, especially considering the complexities of protein networks. In several cases, inclusion of higher-order RS-HDMR component functions significantly improved predictive capability of the model. RS-HDMR is special in its ability to quantitatively capture such cooperativity within an efficient hierarchical framework, without resorting to discretization. Ultimately, sensitivity analysis derived from the resultant RS-HDMR-generated component functions allows for quantitative comparison of the relative interaction strength for each input variable, and significant connectivities can be aggregated to form a general representation of network topology.

High-throughput measurement techniques are becoming more efficient and precise, further transforming biology into a data-driven science/engineering field. Novel analysis techniques such as RS-HDMR are needed to fully utilize these new sources of multivariate data. RS-HDMR can be applied to other biological networks, including transcriptional regulation networks and synthetic gene circuits, as a general tool to quantitatively characterize high-dimensional nonlinear IO interactions. Given appropriate normalization procedures, RS-HDMR can also be used to interpret an amalgamation of data taken from not only different experiments, but from different assays as well. Network identification through RS-HDMR analysis can ultimately be used to direct biological network manipulation and control. This can be achieved by (a) sensitivity analysis of computational models or field data, or (b) direct optimization utilizing the FEOM [30], [74], [77].

## Methods

### RS-HDMR Component Functions

In this work, RS-HDMR component functions are approximated as weighted orthonormal basis functions in order to reduce the sampling effort, and they take the following form:
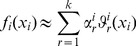
(6)

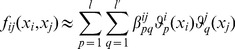
(7)

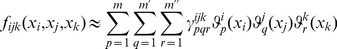
(8)Where 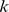,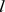,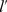,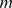,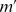,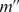 are integers (generally 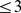 for most applications), 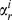, 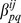, and 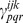 are constant weighting coefficients to be determined, and the basis functions 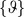 are optimized from the distribution of sample data points to follow conditions of orthonormality [74]. The basis functions are approximated in this work as polynomials, where
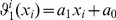
(9)

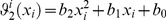
(10)

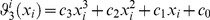
(11)


The coefficients 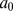,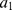,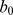,…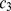 are calculated through monte carlo integration under constraints of orthonormality, such that when integrated over all data points in the training set,
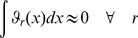
(12)

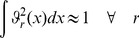
(13)

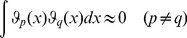
(14)


The weighting coefficient for these basis functions (e.g., 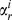 and 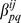 for first and second order component functions, respectively) are then calculated from least-squares fitting to the data.

### RS-HDMR Sensitivity Analysis

An explicit expression is presented here for the sensitivity indices, 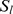, which are used to quantify the relative strength of the network interactions and their respective RS-HDMR component functions. See previous work for a more detailed description [35], [69], [71], [74]. The RS-HDMR expansion may be given in terms of the 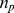 significant component functions 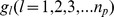, such that.
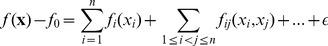
(15)

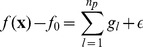
(16)where 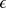 represents any residual error of the model. The total variance, 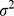, of an output variable 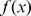 is then defined as follows, summed over all 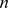 data points:
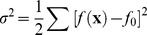
(17)

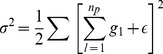
(18)


The RS-HDMR component functions are calculated to be mutually orthogonal when the input variables are sampled independently of one another. However, the orthogonality of distinct component functions may not be strictly upheld under conditions of correlation among input variables. Consequently, the output variance 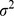 can be decomposed in terms of independent and correlated contributions of the RS-HDMR component functions, where the correlated contributions are described as the summed pairwise-covariances of the individual component functions:
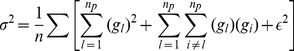
(19)


The sensitivity indices, 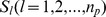, are then defined as the portion of the total variance represented by the 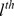 component function out of 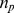 total number of functions [71]. The relationship between sensitivity indices and the output variance 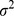 is given as
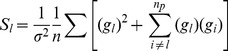
(20)


An output 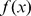 can be described through the RS-HDMR expansion as a hierarchy of first, second, and higher-order RS-HDMR component functions. The total variance of an output 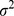 can likewise be decomposed into a hierarchy of sensitivity indices which describe contributions to the total variance from corresponding first, second, and higher-order component functions. The 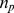 significant component functions and their respective sensitivity indices 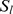 can be explicitly specified as corresponding to particular component functions, such that 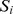 represents the sensitivity index of the RS-HDMR component function 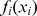. 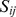 corresponds to the second-order component function 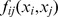. The hierarchical decomposition of the output variance into sensitivity values thus takes the following form:
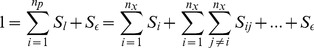
(21)

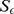
 represents the sensitivity index of the residual variation of the model. The sensitivity indices describing first (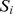), second (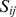), and third order (and higher, if warranted) component functions of input variable 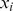 can then be summed into indices, 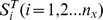, describing both independent and higher-order effects of 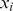 on an output (where 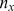 represents the total number of input variables). The magnitudes of 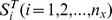 were analyzed to quantify the relative strength of connections between the output variable and the inputs, acting both independently and cooperatively.

Further discussion of the RS-HDMR algorithm and experimental details regarding the single-cell signaling network data can be found in Supplementary Text S1.

### Amelia II and SVM Implementation

Amelia II was downloaded as an R-package and implemented as previously described [37], [38], [60]. We used Amelia II to predict the single-cell values of individual nodes in the T-cell signaling network based on the values of other nodes in the same cell and training data taken from other cells under the same experimental conditions. We implemented an SVM classifier to infer the experimental condition of a single cell based on its measured network state. SVM was performed using the function SVMtrain() in MATLAB (R2009a, The MathWorks, Natick, MA), with a two-norm soft-margin SVM classifier and linear kernel. For both Amelia II and SVM classification, 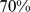 of the data served as a training set to infer the values of the remaining 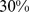 test set of data. We repeated the procedures using different subsets of data as the training set to control for stochastic effects, which ultimately were negligible. The mean values of forty multiple imputations served as the Amelia II predictors of the test set, as described in the previous Dream3 implementation [38].

### Software

A version of RS-HDMR [71] can be found online at http://www.aerodyne.com. It is free for academic users.

## Supporting Information

Figure S1Model IO Network. Weights of the arrows between input and output nodes indicate approximate sensitivity index 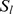 of the respective network connectivity, ranging from 0.60 to 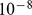. The dashed blue arrow represents the indirect connection (added as a model modification/perturbation) between 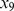 and the output, where 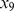 is only related to the output through 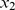. A strong direct connection between 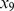 and the output is also added for part of the analysis, shown by the dashed red arrow.(PDF)

Figure S2Heat Map of the Model’s First-Order RS-HDMR Sensitivity Indices. First-order sensitivity indices were derived before RS-HDMR analysis directly from the model. Shown here is a comparison of the calculated RS-HDMR sensitivity indices from the two different algorithms (with and without model reduction) describing the model in Fig. S3. The model was observed under three different conditions (or topologies) to compare the two algorithms’ performance in accurately identifying changes in network topology. In the first model condition (no connection with 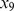), the output is independent of 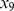. Row 1 describes the sensitivity coefficients of the first model condition calculated directly from the known model coefficients rather than through RS-HDMR inference. Rows 2 and 3 describe RS-HDMR results when the first model condition is observed through uncorrelated, randomly sampled data points. In the second model condition (rows 4 and 5), 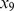 is indirectly connected to the output 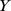 through a correlation with a measured intermediate, 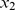. In the third model condition (rows 6 and 7), 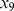 is indirectly related to the output 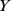, as in the previous condition, but an additional direct connection between 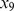 and the output exists.(PDF)

Figure S3Distribution of Calculated Network Connection Sensitivity Indices from Experimental Data. Shown are the cumulative distributions of the RS-HDMR sensitivity indices calculated from two different RS-HDMR algorithms: that with model reduction (MR) and that without (no MR). The indices used are the maximum indices for each network connection observed over the nine individual data sets. Network connections are included in the distribution if they fall above a sensitivity threshold, 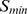, specified on the abscissa.(PDF)

Figure S4 Comparison of RS-HDMR Model Fitting Accuracy. The performance of the two RS-HDMR algorithms was assessed by fitting accuracy. Shown here is a comparison of the fitting accuracy of the two different algorithms (with and without model reduction) as applied to the test model described in Figure S3. Differences in fitting accuracy become most significant under poor sampling conditions, where data is sparse, noisy, and highly correlated. The fitting accuracies were calculated from data describing the model under correlated sampling conditions.
(PDF)

Figure S5RS-HDMR Identified Highly Significant Network Connections. Network connections fall above a sensitivity index threshold of 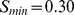. All network connections observed with this level of significance have been previously described in the literature. Black connections are those identified from analysis of individual experimental conditions. Orange network connections describe those identified from pairwise comparison of experimental conditions. The strong connections shown are identified robustly despite conditions of synthetically added noise and truncated sample size (see *Results*).(PDF)

Figure S6RS-HDMR Identified Network Connections, Lower Significance Threshold. Network connections fall above a sensitivity index threshold of 0.05. Connections in blue describe network connections with sensitivity indices falling below the “High Confidence” threshold of 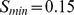, and above the low confidence threshold of 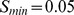. All of these “Low Confidence” connections are unaccounted for in the literature as direct connections.(PDF)

Figure S7Dream4 Network Inference Results. (A) Network structure predicted from the literature [39]. (B) RS-HDMR network inference results. (C) Highly significant RS-HDMR network inference results (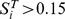). RS-HDMR connections in neither (B) nor (C) were significantly enriched for the literature-based connections in (A), using Fisher’s exact test. The possible reasons are described in the main text.(PDF)

Table S1Test Model Coefficients. The only non-zero second-order coefficient is given by 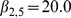. 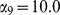 for the model condition with a direct connection to 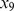.(PDF)

Table S2 Perturbative conditions and their associated effects. Nine total data sets were used in the RS-HDMR analyses, each describing the network under a different perturbative condition, reported in more detail by Sachs et al., 2005. General stimulatory agents (Anti-CD3/CD28) were used to activate T cells and induce proliferation in all but two of the data sets.
(PDF)

Table S3 Previously identified network connections and citations. Network connections identified through RS-HDMR analysis have been described in the previous literature. These interactions are indicated, as well as unmeasured intermediates through which they might occur.
(PDF)

Text S1 Supporting Materials.
(PDF)
