# Supplementary figures and images for: Identifying Biological Network Structure, Predicting Network Behavior, and Classifying Network State With High Dimensional Model Representation (HDMR)
Source: PLoS One. 2012 Jun 18;7(6):e37664. doi: 10.1371/journal.pone.0037664 (PMC3377689; doi:10.1371/journal.pone.0037664)

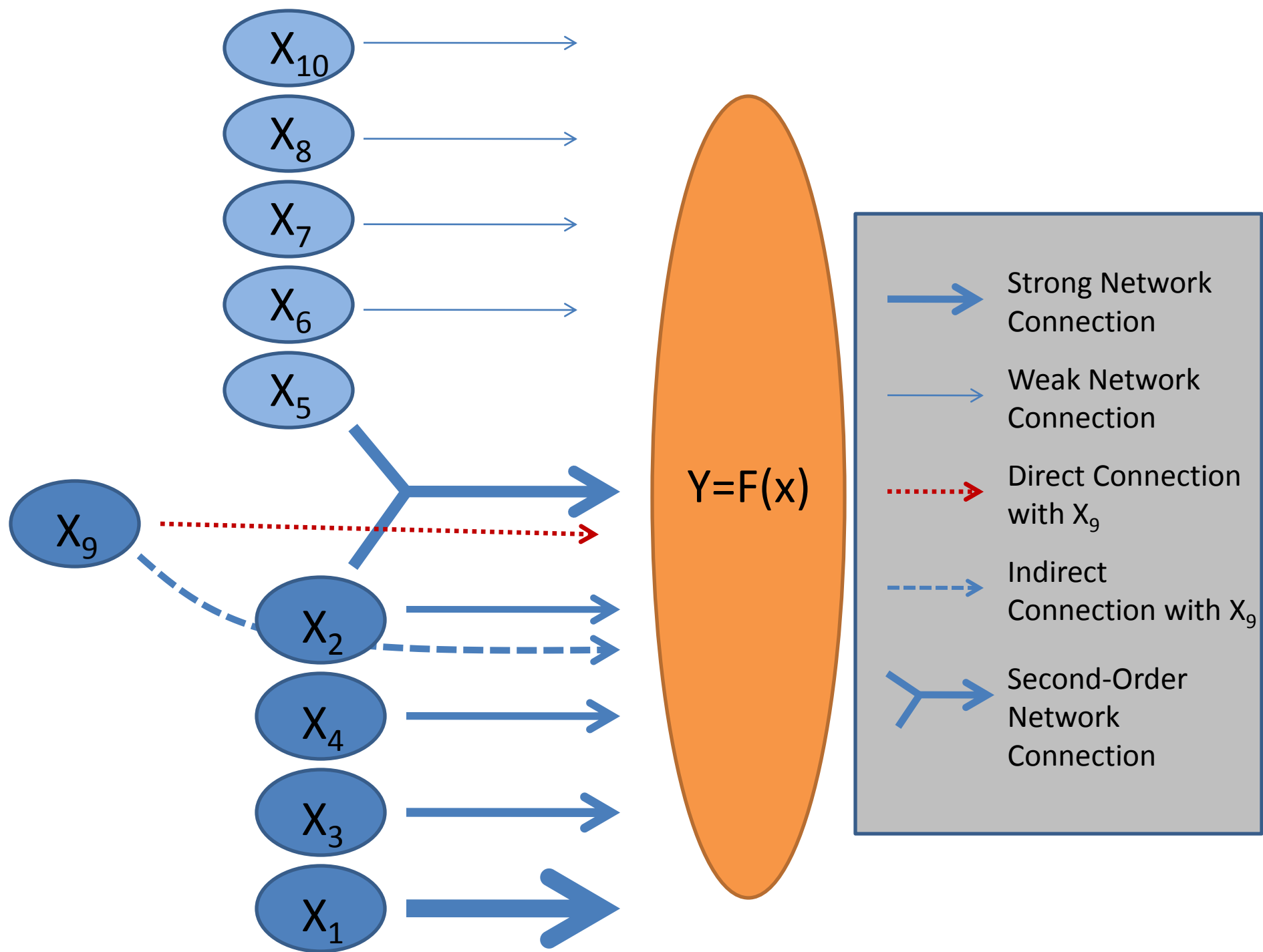

Supplement: Figure S1 — Model IO Network. Weights of the arrows between input and output nodes indicate approximate sensitivity index of the respective network connectivity, ranging from 0.60 to . The dashed blue arrow represents the indirect connection (added as a model modification/perturbation) between and the output, where is only related to the output through . A strong direct connection between and the output is also added for part of the analysis, shown by the dashed red arrow. (PDF) [file pone.0037664.s001.pdf]

Distribution of Sensitivity Indices

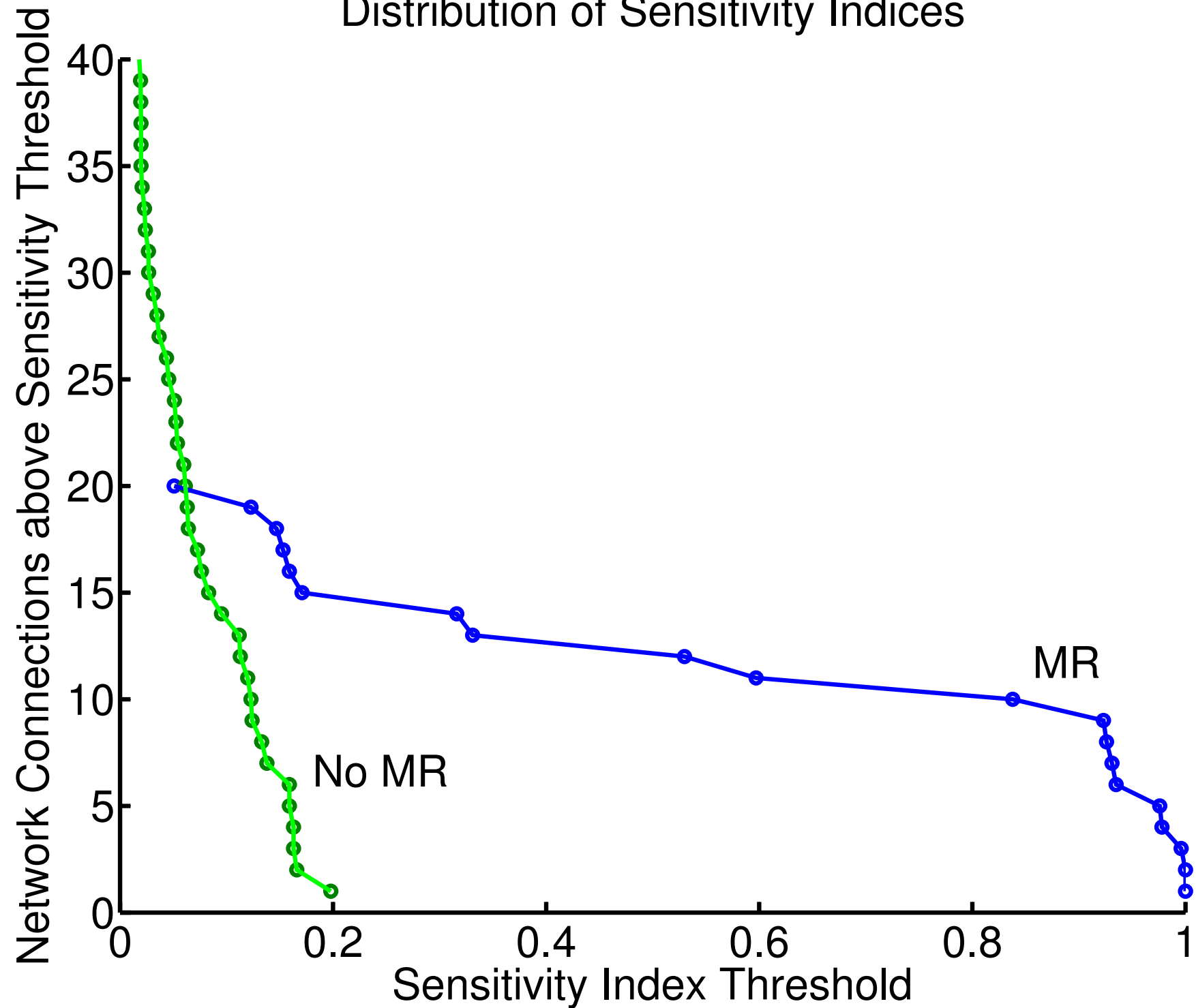

Supplement: Figure S3 — Distribution of Calculated Network Connection Sensitivity Indices from Experimental Data. Shown are the cumulative distributions of the RS-HDMR sensitivity indices calculated from two different RS-HDMR algorithms: that with model reduction (MR) and that without (no MR). The indices used are the maximum indices for each network connection observed over the nine individual data sets. Network connections are included in the distribution if they fall above a sensitivity threshold, , specified on the abscissa. (PDF) [file pone.0037664.s003.pdf]

Without Model Reduction

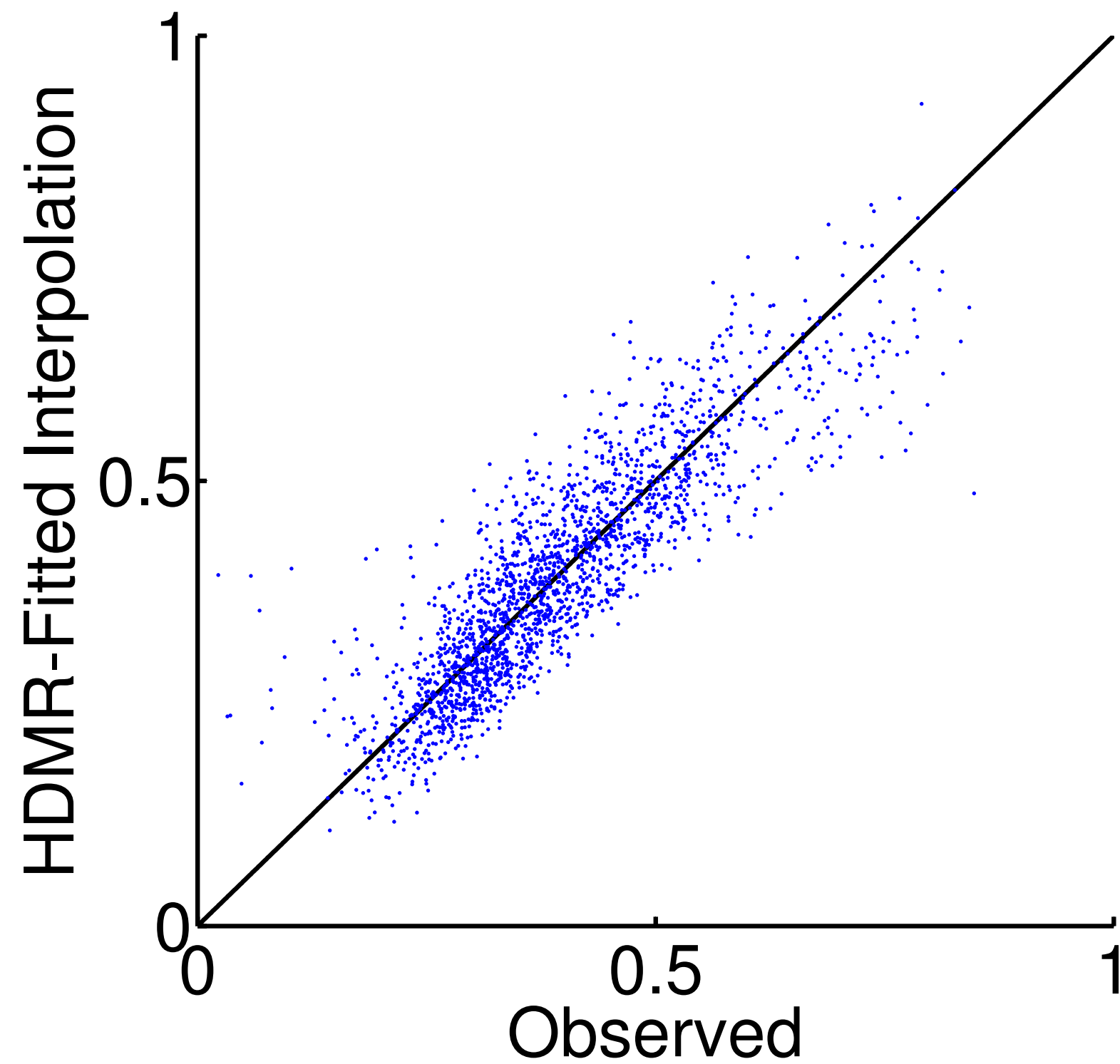

With Model Reduction

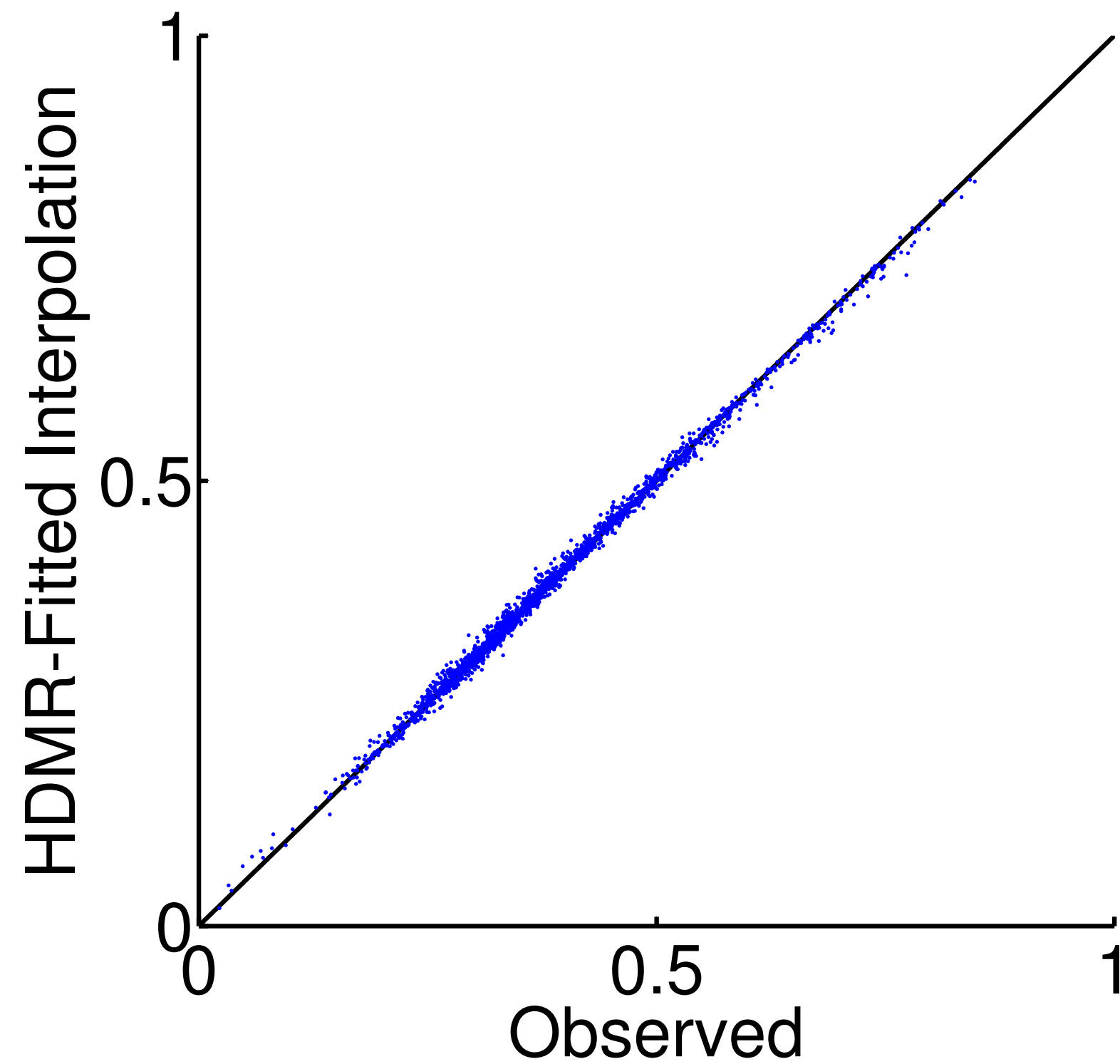

Supplement: Figure S4 — Comparison of RS-HDMR Model Fitting Accuracy. The performance of the two RS-HDMR algorithms was assessed by fitting accuracy. Shown here is a comparison of the fitting accuracy of the two different algorithms (with and without model reduction) as applied to the test model described in Figure S3. Differences in fitting accuracy become most significant under poor sampling conditions, where data is sparse, noisy, and highly correlated. The fitting accuracies were calculated from data describing the model under correlated sampling conditions. (PDF) [file pone.0037664.s004.pdf]

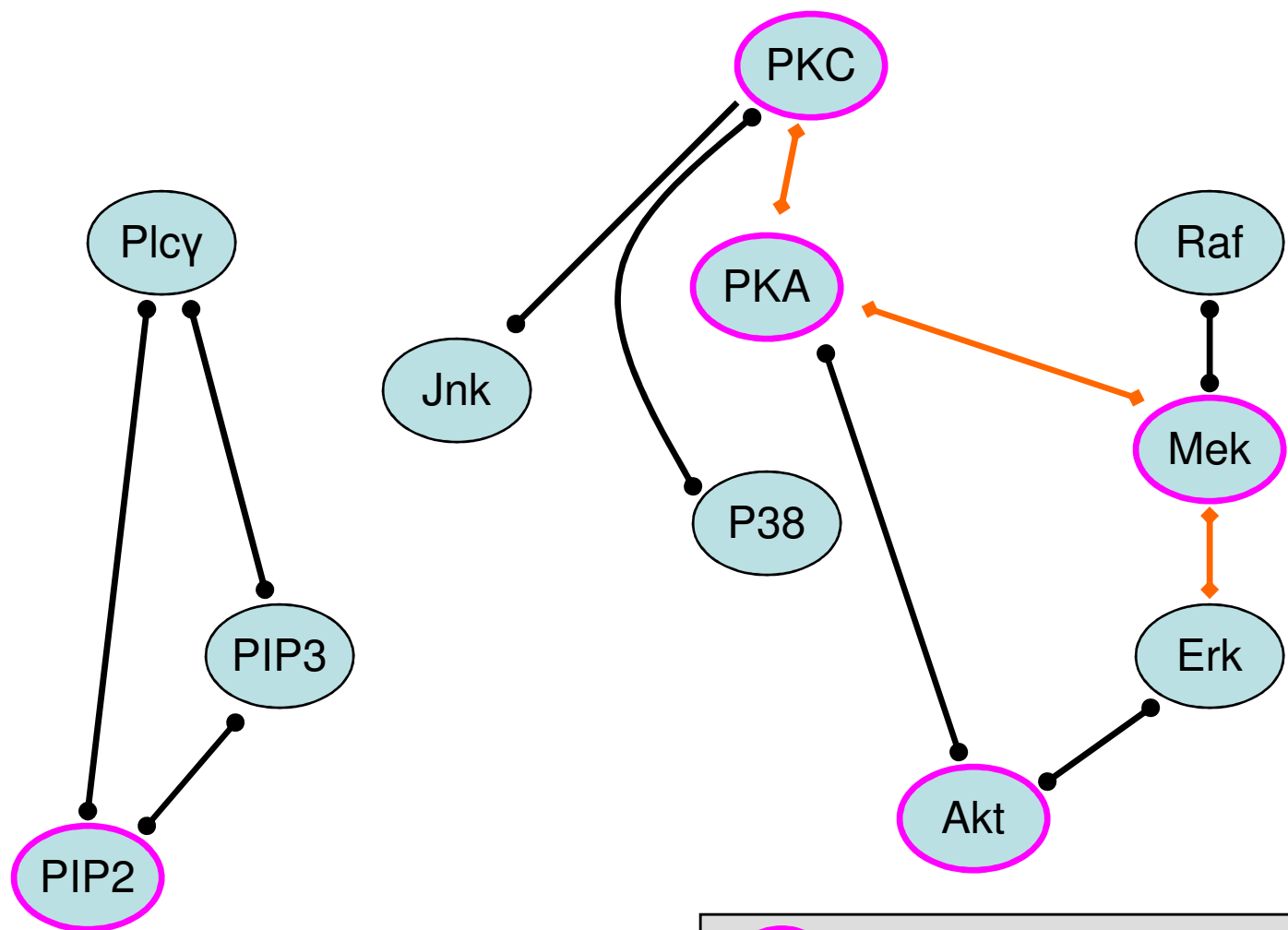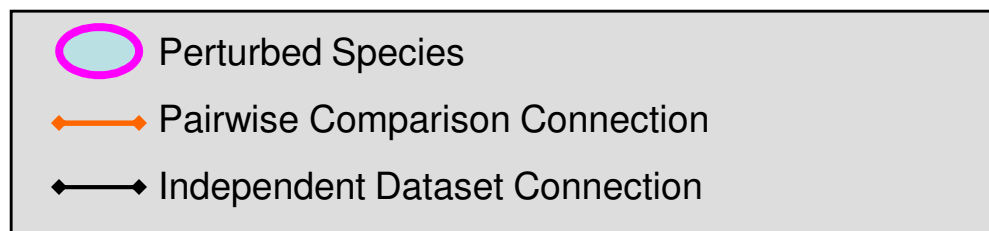

Supplement: Figure S5 — RS-HDMR Identified Highly Significant Network Connections. Network connections fall above a sensitivity index threshold of . All network connections observed with this level of significance have been previously described in the literature. Black connections are those identified from analysis of individual experimental conditions. Orange network connections describe those identified from pairwise comparison of experimental conditions. The strong connections shown are identified robustly despite conditions of synthetically added noise and truncated sample size (see Results). (PDF) [file pone.0037664.s005.pdf]

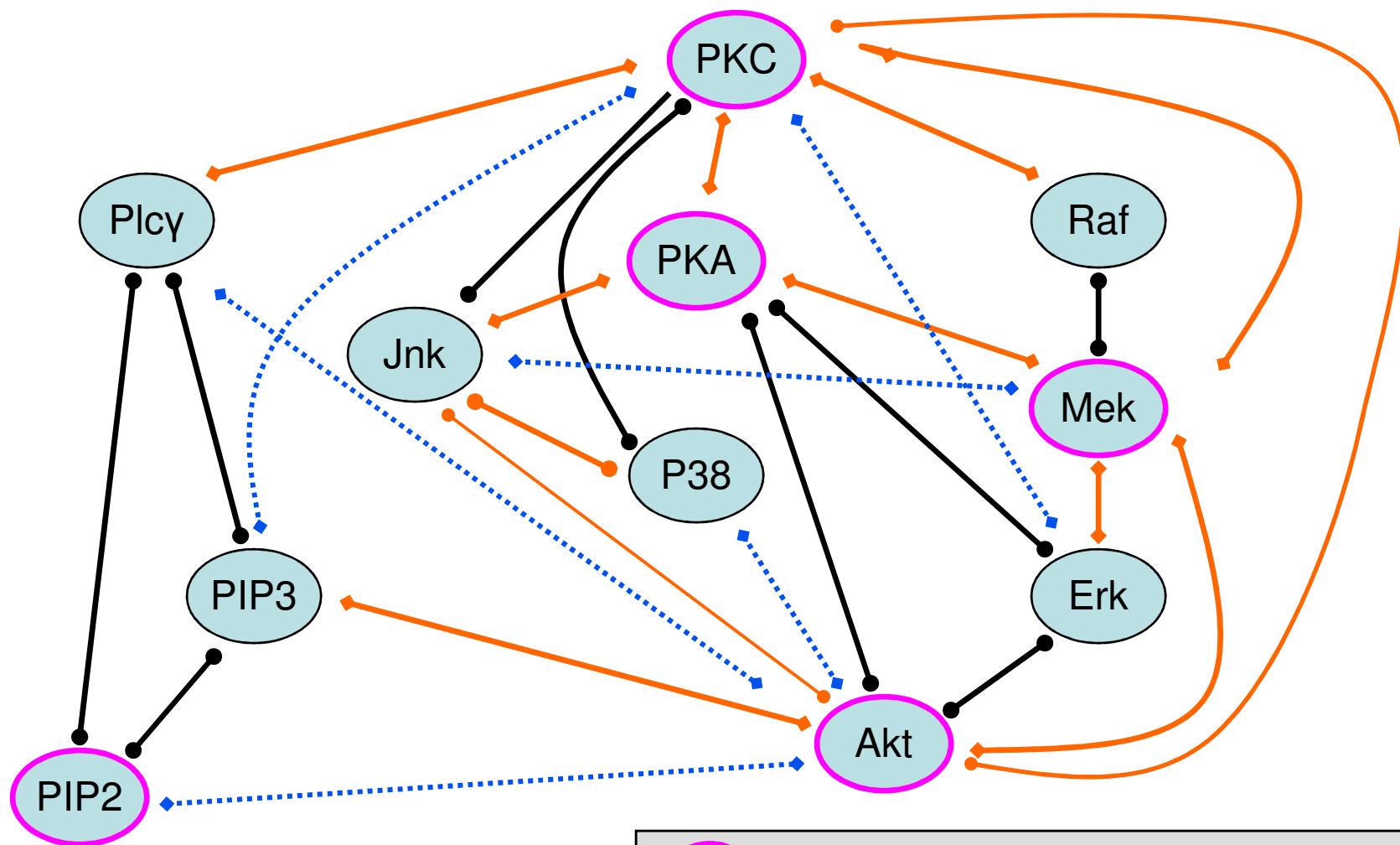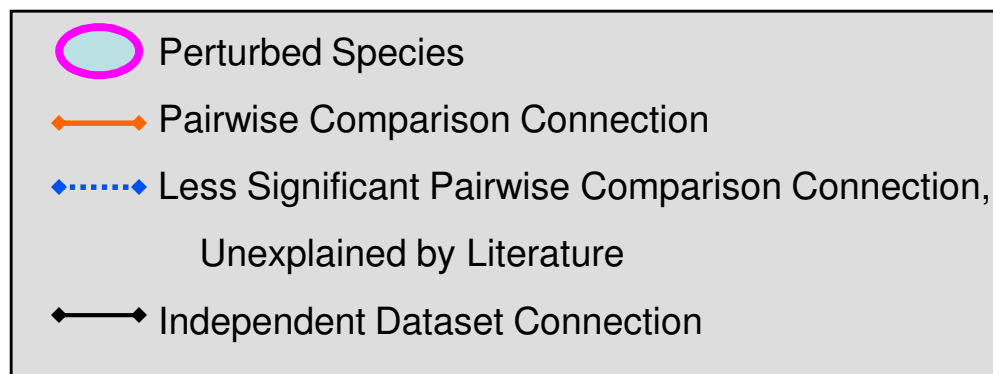

Supplement: Figure S6 — RS-HDMR Identified Network Connections, Lower Significance Threshold. Network connections fall above a sensitivity index threshold of 0.05. Connections in blue describe network connections with sensitivity indices falling below the “High Confidence” threshold of , and above the low confidence threshold of . All of these “Low Confidence” connections are unaccounted for in the literature as direct connections. (PDF) [file pone.0037664.s006.pdf]

A

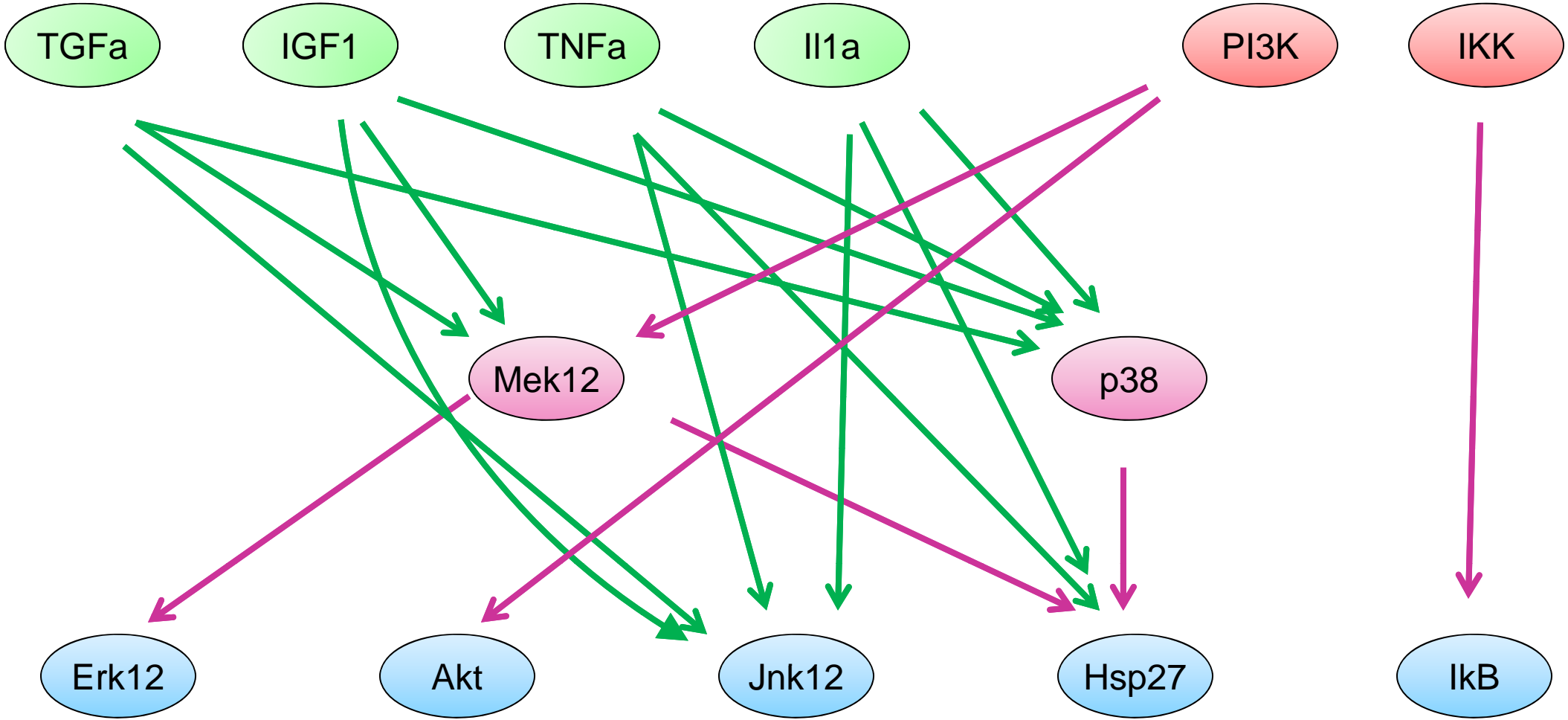

B

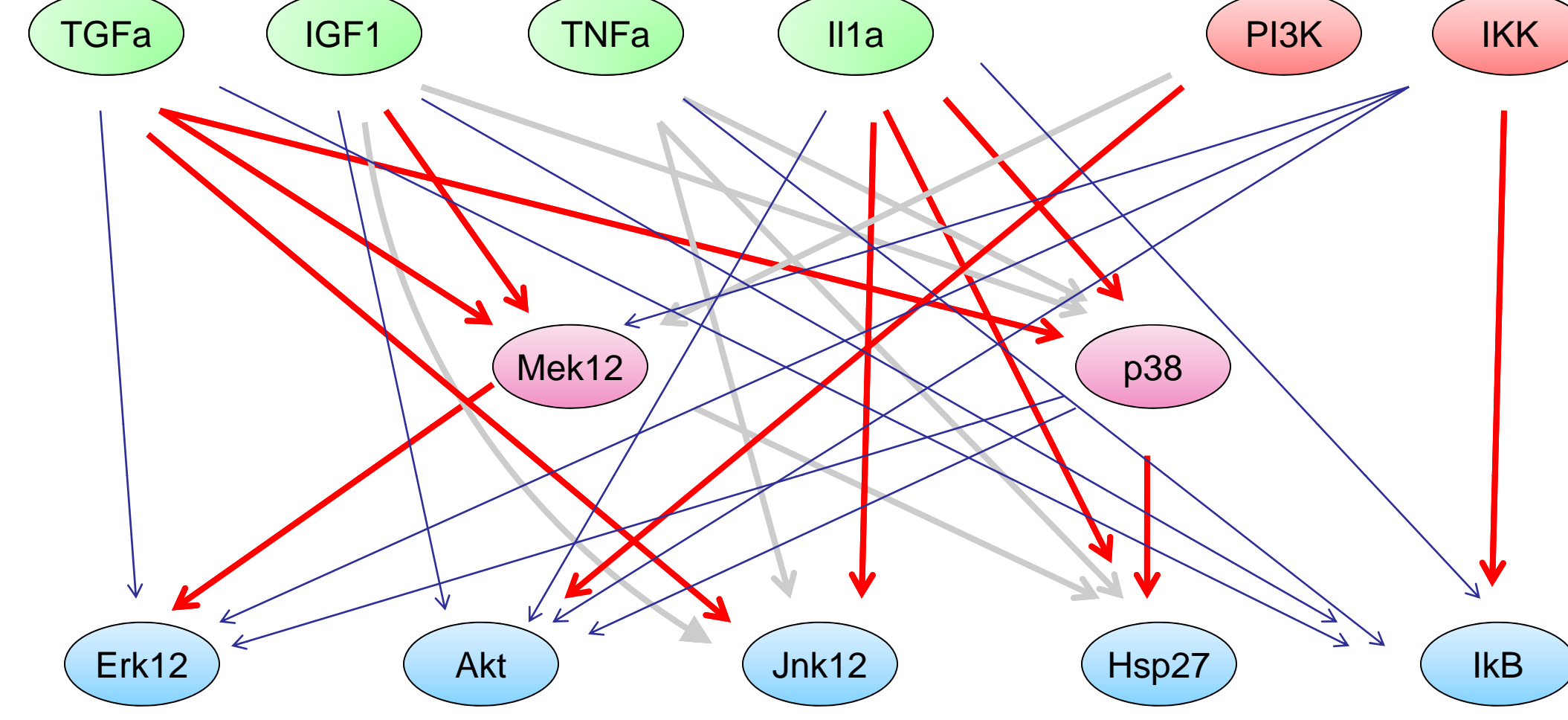

C

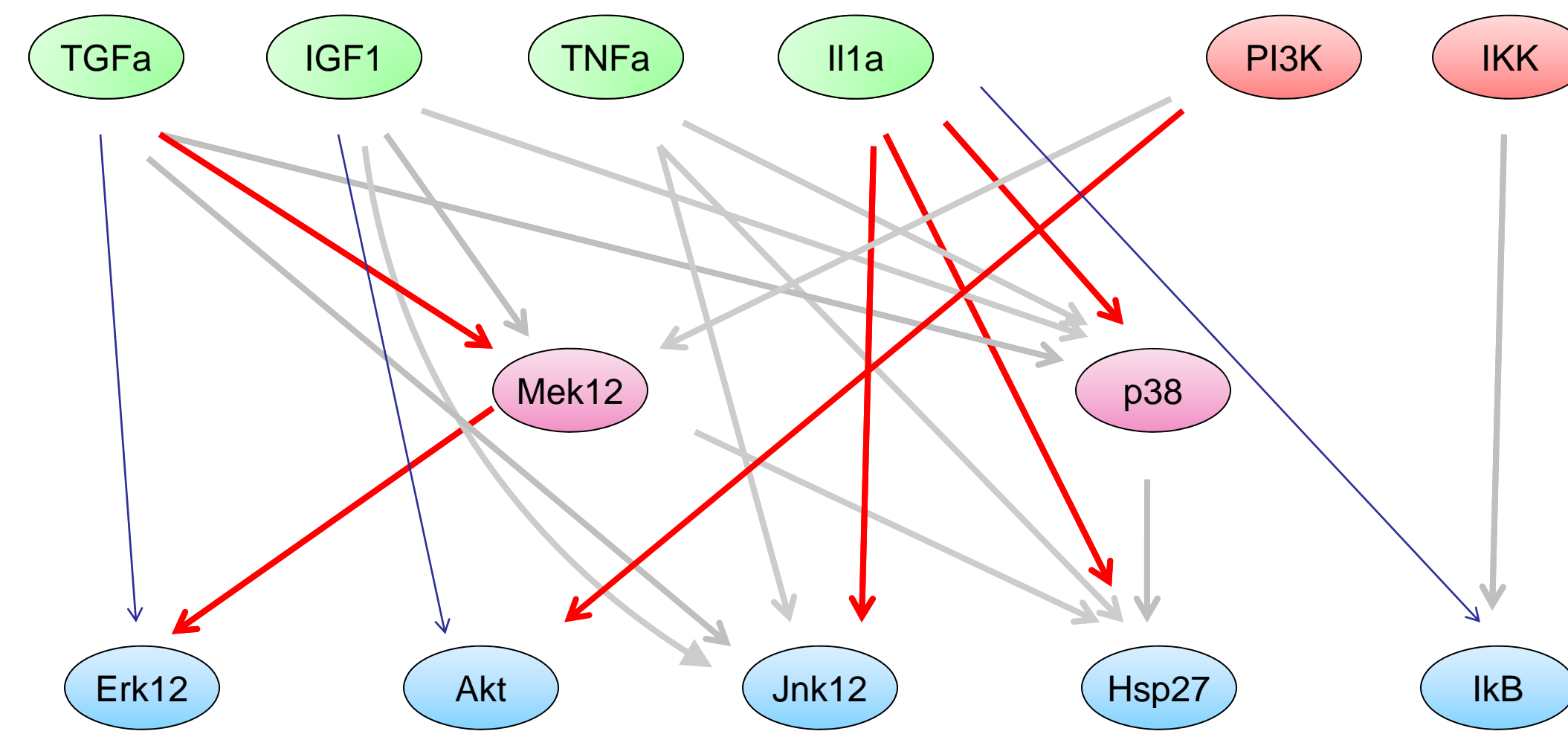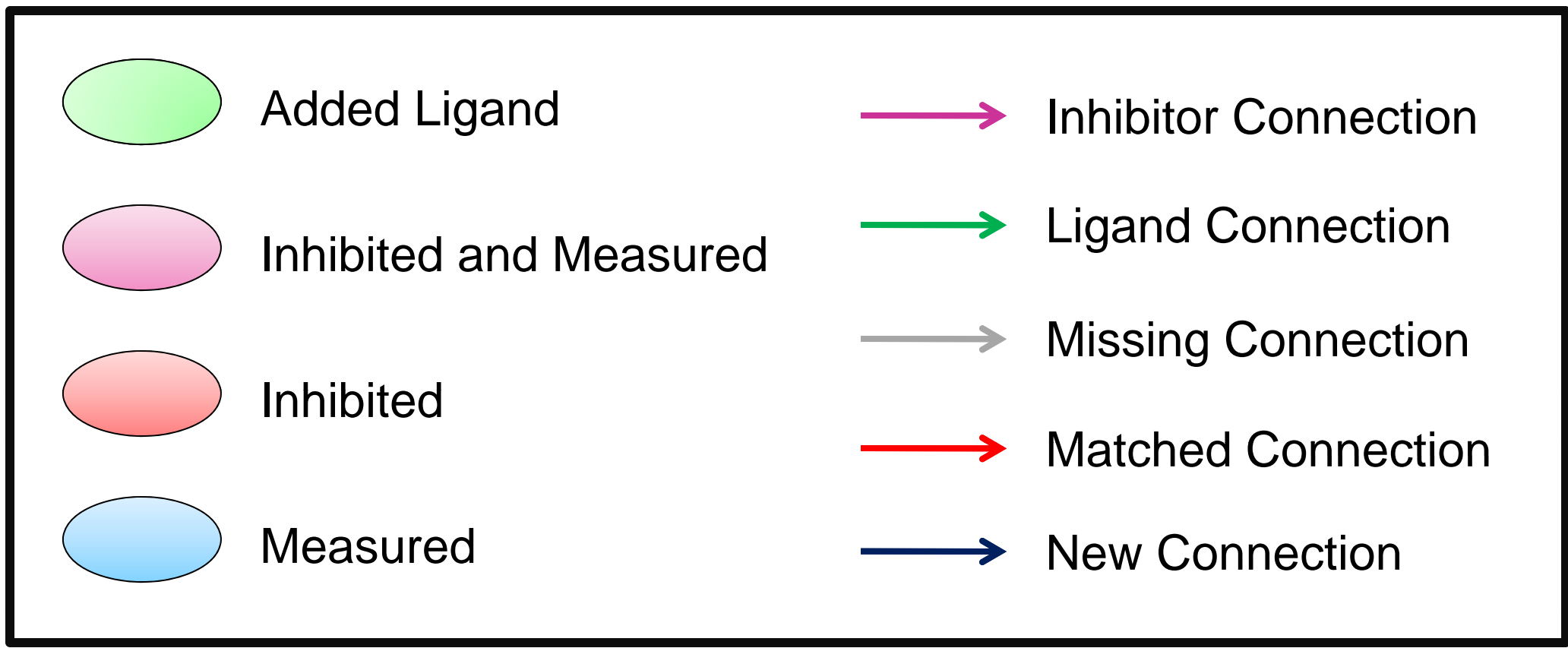

Supplement: Figure S7 — Dream4 Network Inference Results. (A) Network structure predicted from the literature [39]. (B) RS-HDMR network inference results. (C) Highly significant RS-HDMR network inference results (). RS-HDMR connections in neither (B) nor (C) were significantly enriched for the literature-based connections in (A), using Fisher’s exact test. The possible reasons are described in the main text. (PDF) [file pone.0037664.s007.pdf]
