# Supplementary material for: Identifying Biological Network Structure, Predicting Network Behavior, and Classifying Network State With High Dimensional Model Representation (HDMR)
Source: PLoS One. 2012 Jun 18;7(6):e37664. doi: 10.1371/journal.pone.0037664 (PMC3377689; doi:10.1371/journal.pone.0037664)
Supplement: Figure S2 — Heat Map of the Model’s First-Order RS-HDMR Sensitivity Indices. First-order sensitivity indices were derived before RS-HDMR analysis directly from the model. Shown here is a comparison of the calculated RS-HDMR sensitivity indices from the two different algorithms (with and without model reduction) describing the model in Fig. S3. The model was observed under three different conditions (or topologies) to compare the two algorithms’ performance in accurately identifying changes in network topology. In the first model condition (no connection with ), the output is independent of . Row 1 describes the sensitivity coefficients of the first model condition calculated directly from the known model coefficients rather than through RS-HDMR inference. Rows 2 and 3 describe RS-HDMR results when the first model condition is observed through uncorrelated, randomly sampled data points. In the second model condition (rows 4 and 5), is indirectly connected to the output through a correlation with a measured intermediate, . In the third model condition (rows 6 and 7), is indirectly related to the output , as in the previous condition, but an additional direct connection between and the output exists. (PDF) [file pone.0037664.s002.pdf]

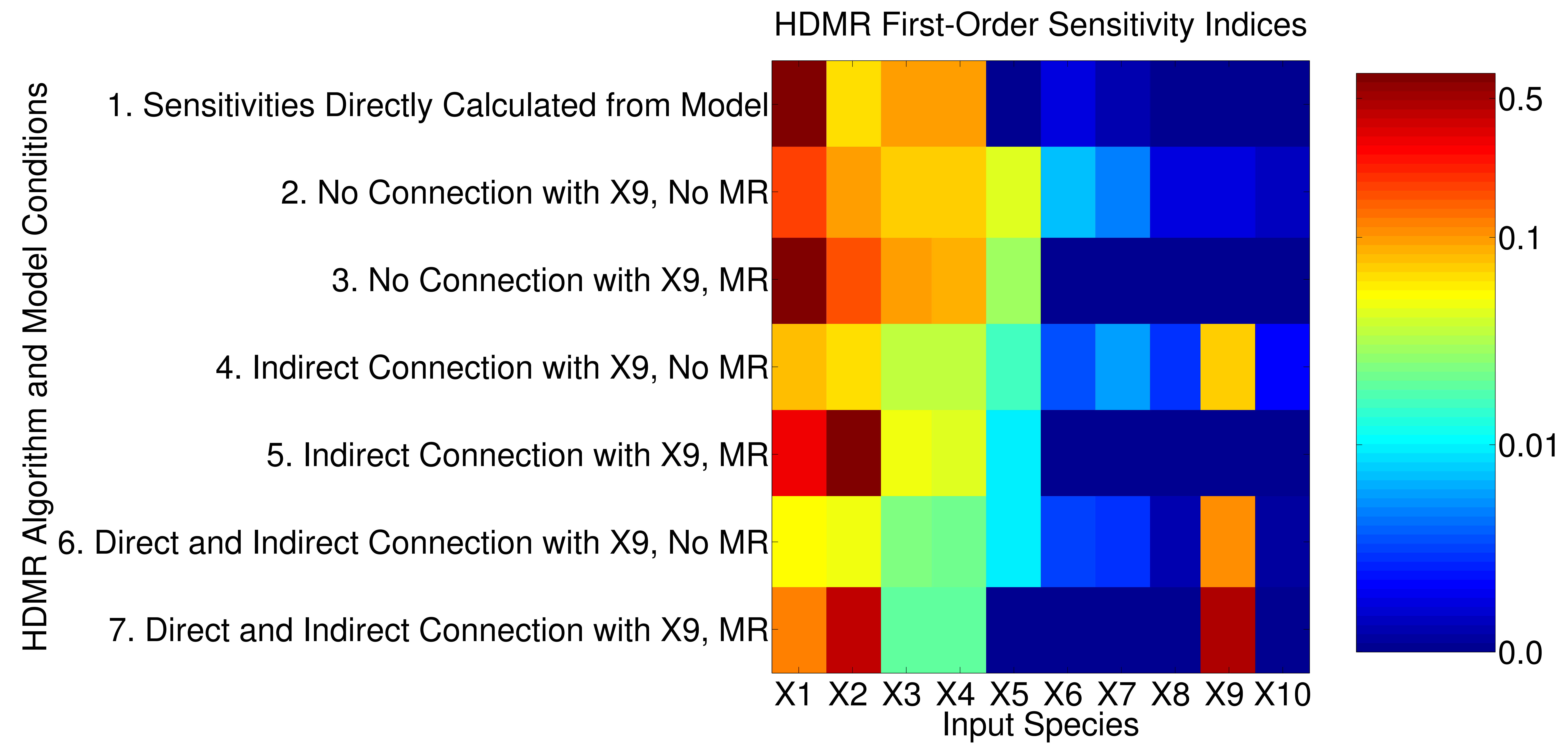
HDMR First-Order Sensitivity Indices
HDMR Algorithm and Model Conditions
1. Sensitivities Directly Calculated from Model
2. No Connection with X9, No MR
3. No Connection with X9, MR
4. Indirect Connection with X9, No MR
5. Indirect Connection with X9, MR
6. Direct and Indirect Connection with X9, No MR
7. Direct and Indirect Connection with X9, MR
X1 X2 X3 X4 X5 X6 X7 X8 X9 X10
Input Species
0.5
0.1
0.01
0.0
