# Supplementary material for: Identifying Biological Network Structure, Predicting Network Behavior, and Classifying Network State With High Dimensional Model Representation (HDMR)
Source: PLoS One. 2012 Jun 18;7(6):e37664. doi: 10.1371/journal.pone.0037664 (PMC3377689; doi:10.1371/journal.pone.0037664)
Supplement: Table S1 — Test Model Coefficients. The only non-zero second-order coefficient is given by . for the model condition with a direct connection to . (PDF) [file pone.0037664.s008.pdf]

Table S1: Test Model Coefficients.

i	α_i	a_i	b_i	c_i
1	40.0	3	0	1
2	10.0	2	2	1
3	10.0	-3	-4	1
4	20.0	-1	-1	-1
5	0.1	5	3	0.1
6	0.6	7	7	1
7	0.5	3	1.5	2
8	0.05	1	2	3
9	0.0	3	1	1
10	0.01	2	1	2

The only non-zero second-order coefficient is given by $\beta_{2,5} = 20.0$. $\alpha_9 = 10.0$ for the model condition with a direct connection to x_9.
