# Supplementary material for: Identifying Biological Network Structure, Predicting Network Behavior, and Classifying Network State With High Dimensional Model Representation (HDMR)
Source: PLoS One. 2012 Jun 18;7(6):e37664. doi: 10.1371/journal.pone.0037664 (PMC3377689; doi:10.1371/journal.pone.0037664)
Supplement: Table S2 — Perturbative conditions and their associated effects. Nine total data sets were used in the RS-HDMR analyses, each describing the network under a different perturbative condition, reported in more detail by Sachs et al., 2005. General stimulatory agents (Anti-CD3/CD28) were used to activate T cells and induce proliferation in all but two of the data sets. (PDF) [file pone.0037664.s009.pdf]

Table S2: Perturbative conditions and their associated effects.

Data set Employed	Reagent	Biological Effect
d_1-d_7	Anti-CD3/CD28	General T-cell activation
d_2	ICAM-2	General Signaling Activation
d_3	AKT Inhibitor	AKT Inhibition
d_4	G06976	PKC Inhibition
d_5	Psitectorigenin	PIP2 Inhibition
d_6	U0126	Mek1, Mek2 Inhibition
d_7	LY294002	Akt Inhibition
d_8	PMA	PKC activation
d_9	β2cAMP	PKA activation

Nine total data sets were used in the RS-HDMR analyses, each describing the network under a different perturbative condition, reported in more detail by Sachs et al., 2005. General stimulatory agents (Anti-CD3/CD28) were used to activate T cells and induce proliferation in all but two of the data sets.
