# Supplementary material for: Identifying Biological Network Structure, Predicting Network Behavior, and Classifying Network State With High Dimensional Model Representation (HDMR)
Source: PLoS One. 2012 Jun 18;7(6):e37664. doi: 10.1371/journal.pone.0037664 (PMC3377689; doi:10.1371/journal.pone.0037664)
Supplement: Table S3 — Previously identified network connections and citations. Network connections identified through RS-HDMR analysis have been described in the previous literature. These interactions are indicated, as well as unmeasured intermediates through which they might occur. (PDF) [file pone.0037664.s010.pdf]

Table S3: Previously identified network connections and citations.

Connection	Influence Path	Citation
PKC → Raf	PKC → *Ras* → Raf_{S259}	[1, 2]
PKC → Mek	PKC → $Ras_{S497/S499}$ → Mek	[3]
PKC → Jnk	PKC → → *MKKs* → Jnk	[4]
PKC → p38	PKC → → *MKKs* → p38	[4]
PKC → PKA	PKC → *cAMP* → PKA	[5]
PKA → Raf	PKA → Raf_{S259}	[6]
PKA → Mek	PKA → Raf_{S621} → Mek	[7]
PKA → Erk	PKA → *HePTP* → Erk	[8]
PKA → Jnk	PKA → → *MKKs* → Jnk	[9]
PKA → p38	PKA → → *MKKs* → p38	[10]
Raf → Mek	Direct Phosphorylation	[11]
PKA → Akt	PKA → *CaMKK* → Akt_{T308} → Akt_{S473}	[12, 13]
Mek → Erk	Direct Phosphorylation	[14]
Plcγ → PIP3	Direct Phosphorylation	[15, 16]
Plcγ → PIP2	Recruitment leading to phosphorylation	[17]
Plcγ → PKC	PLC → DAG → PKC	[18–20]
PIP3 → PIP2	Precursor-Product	[17]
PIP3 → Akt	PIP3 → Akt_{T308} → Akt_{S473}	[21, 22]
PIP2 → PKC	PIP2 → DAG → PKC	[18–20]
Erk → Akt	direct or indirect	[23]
Akt → Jnk	Akt → JIP1 → Jnk	[24, 25]
Akt → PKC	Akt → PH domain association	[26, 27]
Akt → Mek	Akt → Raf_{S338} → Mek	[28]
Jnk → p38	Jnk ← *MKKs* → p38	[29, 30]

Network connections identified through RS-HDMR analysis have been described in the previous literature. These interactions are indicated, as well as unmeasured intermediates through which they might occur.

References

[1] Van Aelst L, Barr M, Marcus S, Polverino A, Wigler M (1993) Complex formation between RAS and RAF and other protein kinases. Proc Natl Acad Sci US A 90: 6213–7.

[2] Marais R, Light Y, Mason C, Paterson H, Olson M, et al. (1998) Requirement of Ras-GTP-Raf Complexes for Activation of Raf-1 by Protein Kinase C. Science 280: 109.

[3] Carroll M, May W (1994) Protein kinase C-mediated serine phosphorylation directly activates Raf-1 in murine hematopoietic cells. J Biol Chem 269: 1249–1256.

[4] Clerk A, Pham F, Fuller S, Sahai E, Aktories K, et al. (2001) Regulation of Mitogen-Activated Protein Kinases in Cardiac Myocytes through the Small G Protein Rac1. Mol Cell Biol 21: 1173.

[5] Zhang W, Wong T (1998) Suppression of cyclic AMP by phosphoinositol/Ca 2 pathway in the cardiac-opioid receptor. Am J Physiol Cell Physiol 274: C82–C87.

[6] Dhillon A, Pollock C, Steen H, Shaw P, Mischak H, et al. (2002) Cyclic AMP-Dependent Kinase Regulates Raf-1 Kinase Mainly by Phosphorylation of Serine 259. Mol Cell Biol 22: 3237.

[7] Mischak H, Seitz T, Janosch P, Eulitz M, Steen H, et al. (1996) Negative regulation of Raf-1 by phosphorylation of serine 621. Mol Cell Biol 16: 5409.

[8] Blanco-Aparicio C, Torres J, Pulido R (1999) A Novel Regulatory Mechanism of MAP Kinases Activation and Nuclear Translocation Mediated by PKA and the PTP-SL Tyrosine Phosphatase. J Cell Biol 147: 1129–1136.

[9] Fortino V, Torricelli C, Gardi C, Valacchi G, Rossi Paccani S, et al. (2002) ERKs are the point of divergence of PKA and PKC activation by PTHrP in human skin fibroblasts. Cell Mol Life Sci 59: 2165–2171.

[10] Zheng M, Zhang S, Zhu W, Ziman B, Kobilka B, et al. (2000) β2-Adrenergic Receptor-induced p38 MAPK Activation Is Mediated by Protein Kinase A Rather than by Gi or G$\beta\gamma$ in Adult Mouse Cardiomyocytes. J Biol Chem 275: 40635–40640.

[11] Lange-Carter C, Johnson G (1994) Ras-dependent growth factor regulation of MEK kinase in PC12 cells. Science 265: 1458–1461.

[12] Wayman G, Tokumitsu H, Soderling T (1997) Inhibitory Cross-talk by cAMP Kinase on the Calmodulin-dependent Protein Kinase Cascade. J Biol Chem 272: 16073–16076.

[13] Yano S, Tokumitsu H, Soderling T (1998) Calcium promotes cell survival through CaM-K kinase activation of the protein-kinase-B pathway. Nature 396: 584–587.

[14] Payne D, Rossomando A, Martino P, Erickson A, Her J, et al. (1991) Identification of the regulatory phosphorylation sites in pp42/mitogen-activated protein kinase (MAP kinase). EMBO J 10: 885–892.

[15] Sofroniew M, Howe C, Mobley W (2001) Nerve growth factor signaling, neuroprotection, and neural repair. Annu Rev Neurosci 24: 1217–1281.

[16] Lee S, Rhee S (1995) Significance of PIP, hydrolysis and regulation of phospholipase C isozymes. Curr Opin Cell Biol 7: 183–189.

[17] Czech M (2000) PIP2 and PIP3 Complex Roles at the Cell Surface. Cell 100: 603–606.

[18] Noh D, Shin S, Rhee S (1995) Phosphoinositide-specific phospholipase C and mitogenic signaling. BBA Lib 1242: 99–113.

[19] Brown K, Blay J, Irvine R, Heslop J, Berridge M (1984) Reduction of epidermal growth factor receptor affinity by heterologous ligands: evidence for a mechanism involving the breakdown of phosphoinositides and the activation of protein kinase C. Biochem Biophys Res Commun 123: 377–84.

[20] Rana R, Hokin L (1990) Role of phosphoinositides in transmembrane signaling. Physiol Rev 70: 115–164.

[21] Alessi D, James S, Downes C, Holmes A, Gaffney P, et al. (1997) Characterization of a 3-phosphoinositide-dependent protein kinase which phosphorylates and activates protein kinase Balpha. Curr Biol 7: 261–269.

[22] Stokoe D, Stephens L, Copeland T, Gaffney P, Reese C, et al. (1997) Dual Role of Phosphatidylinositol-3, 4, 5-trisphosphate in the Activation of Protein Kinase B. Science 277: 567.

[23] Fukuda R, Kelly B, Semenza G (2003) Vascular Endothelial Growth Factor Gene Expression in Colon Cancer Cells Exposed to Prostaglandin E2 Is Mediated by Hypoxia-inducible Factor 1 1. Cancer Res 63: 2330–2334.

[24] Go Y, Boo Y, Park H, Maland M, Patel R, et al. (2001) Protein kinase B/Akt activates c-Jun NH2-terminal kinase by increasing NO production in response to shear stress. J App Physiol 91: 1574–1581.

[25] Kim A, Yano H, Cho H, Meyer D, Monks B, et al. (2002) Akt1 Regulates a JNK Scaffold during Excitotoxic Apoptosis. Neuron 35: 697–709.

[26] Greco S, Storelli C, Marsigliante S (2006) Protein kinase C (PKC)-δ/-ε mediate the PKC/Akt-dependent phosphorylation of extracellular signal-regulated kinases 1 and 2 in MCF-7 cells stimulated by bradykinin. J Endocrinol 188: 79–89.

[27] Konishi H, Matsuzaki H, Tanaka M, Ono Y, Tokunaga C, et al. (1996) Activation of RAC-protein kinase by heat shock and hyperosmolarity stress through a pathway independent of phosphatidylinositol 3-kinase. Proc Natl Acad Sci US A 93: 7639–7643.

[28] Rommel C, Clarke B, Zimmermann S, Nuñez L, Rossman R, et al. Differentiation Stage-Specific Inhibition of the Raf-MEK-ERK Pathway by Akt. Science 286: 1738–1741.

[29] Deacon K, Blank J (1997) Characterization of the Mitogen-activated Protein Kinase Kinase 4 (MKK4)/c-Jun NH2-terminal kinase 1 and MKK3/p38 Pathways Regulated by MEK Kinases 2 and 3 Mek Kinase 3 activates MKK3 but does not cause activation of p38 kinase in vivo. J Biol Chem 272: 14489–14496.

[30] Guan Z, Buckman S, Pentland A, Templeton D, Morrison A (1998) Induction of Cyclooxygenase-2 by the Activated MEKK1 – SEK1/MKK4 – p38 Mitogen-activated Protein Kinase Pathway. J Biol Chem 273: 12901–12908.
