# Supplementary material for: Identifying Biological Network Structure, Predicting Network Behavior, and Classifying Network State With High Dimensional Model Representation (HDMR)
Source: PLoS One. 2012 Jun 18;7(6):e37664. doi: 10.1371/journal.pone.0037664 (PMC3377689; doi:10.1371/journal.pone.0037664)
Supplement: Text S1 — Supporting Materials. (PDF) [file pone.0037664.s011.pdf]

Supporting Text S1

1 RS-HDMR Algorithmic Modifications

High levels of noise, low sample size, and highly correlated variables are frequent problems when dealing with experimental data describing biological systems. These issues restrict the ability to identify exact IO relationships, particularly with respect to higher order cooperative interactions. Consequently, several algorithmic adjustments of RS-HDMR are made to address such ill-posed problems. RS-HDMR component functions extending above third-order are commonly insignificant in applications to most natural systems, especially those described by noisy data and sparse sampling coverage [1]. For this application, only first, second, and third-order RS-HDMR component functions were determined. The component functions are approximated as optimally weighted orthonormal polynomial expansions, with coefficients calculated simultaneously through least-squares regression [2]. Finally, a model reduction (MR) method is employed, such that only input variables which significantly increase the RS-HDMR fitting quality, as measured through a statistical F-test, are included in the calculation of the RS-HDMR IO-mappings [3]. These modifications present RS-HDMR within a computationally tractable framework and strengthen the robustness of the results with respect to high levels of noise and correlation among the input variables, thereby reducing problems of overfitting.

A close relation exists between the problems of addressing strong correlation amongst the input variables and discerning significant versus insignificant network connections in network identification. Correlation amongst the input variables that are tightly interconnected can arise and often leads to skewed and inflated sensitivity analysis results. In addition, distinguishing between significant and insignificant IO connections becomes increasingly difficult when the input variables are strongly correlated. The MR modification

to the RS-HDMR algorithm specifically addresses these issues. Here we apply RS-HDMR analysis with and without MR to a simple IO model that is subject to various sampling and structural perturbations to examine the effect of MR in addressing ill-posed problems typical with biological systems.

1. Model Overview. The model consists of ten inputs, $\mathbf{x} = x_1, x_2, ...x_n (n = 10)$ (Fig. S1). The inputs affect a single output $y = f(\mathbf{x})$ through independent and cooperative non-linear IO connections. For clear interpretation of the RS-HDMR analysis results, these IO connections take a form similar to the non-linear polynomials that the RS-HDMR algorithm approximates such IO connections as having. The model has the following form, where $\alpha_i, \beta_{ij}, a_i, b_i$, and c_i are constants defined in Table S1:

$$f(\mathbf{x}) = f_0 + \sum_{i=1}^{n} \alpha_i (a_i x^3 + b_i x_i^2 + c_i x_i) + \sum_{1 \leq i < j \leq n} \beta_{ij} (a_i x_i^3 + b_i x_i^2 + c_i x_i)(a_j x_j^3 + b_j x_j^2 + c_j x_j) \quad (1)$$

A priori knowledge of the model and its coefficients allows for exact calculation of the corresponding HDMR component function sensitivity indices, without resorting to sampling and Monte-Carlo integration. These calculated sensitivity indices are shown in Fig. S2 and compared to the results calculated from various RS-HDMR algorithms and sampling efforts described below.

2. Weighted Network Connectivity. IO network interactions of various significance were used in the model in order to analyze the RS-HDMR algorithm's ability to identify and discern strong network connections from insignificant ones. The strength of network connectivities were characterized before the RS-HDMR analysis through direct calculation of sensitivity indices from the model (Fig. S2). The four inputs $(x_1 - x_4)$ have relatively

strong first-order interaction with the output, with sensitivity indices all lying above 0.05. Sensitivity indices for the first-order interactions of the remaining six inputs ($x_5 - x_{10}$) all fall below 0.001. The model contains one strong second-order IO interaction between x_2 and x_5, which has a corresponding sensitivity index of 0.13.

3. Direct and Indirect Connections with x_9. We modified the topology of the model to include indirect or both direct and indirect IO interactions between x_9 and the output in order to assess the RS-HDMR algorithm's ability to identify direct (rather than indirect) network connections as significant (Fig. S2). For the indirect connection, x_9 is sampled as a function of x_2 (using either a bivariate normal distribution or correlated Latin hypercube sampling), which has strong direct connection to the output. Thus x_9 is only related to the output through x_2. As another topological modification, a strong direct connection between x_9 and the output is added for comparative purposes. This added IO connection follows the general non-linear polynomial form described in Eq. 1. Results of the RS-HDMR analysis describing various network topologies may then be compared in order to further ascertain if RS-HDMR can distinguish between direct and indirect IO connections.

Sample sizes of 7000 data points were used in the RS-HDMR analysis of the model under all conditions. Input data points were generated from a uniform distribution between 0 and 1. Outliers falling more than two standard deviations from the mean were excluded from analyzed data sets.

2 Results from Model Analysis

1. Discerning between Direct and Indirect IO Connections. RS-HDMR sensitivity analysis, both with and without MR, was performed using data describing the model under several topological perturbations. As an initial control, the model was first observed under good sampling conditions, described by uncorrelated, randomly sampled data points.

Under these conditions, both algorithms produce relatively similar results. However, results from the RS-HDMR sensitivity analyses diverge between the algorithms, with and without MR, upon introduction of significant correlation (or multi-collinearity) amongst the sampled data points. Under the control conditions, neither version of RS-HDMR (with or without MR) identified x_9 to be a significant input variable. Under conditions of added correlation, however, RS-HDMR without MR describes first-order connection with x_9 as significant. In contrast, RS-HDMR with MR succeeds in identifying the relationship as indirect. As a test of positive selection, a strong direct connection between x_9 and the output was added to the model in addition to the indirect connection with x_9. Both RS-HDMR, with and without MR, are successful in capturing x_9 as a significant input.

Biased sensitivity analysis, arising from correlated input variables and overfitting, can also be observed through analysis of the distributions of the sensitivity indices. In general, RS-HDMR without MR (as compared to RS-HDMR with MR) tends to essentially "flatten" the distribution of sensitivity indices for a given IO model. The observed range and variance of the sensitivity indices of the component functions in a given model decreases by overfitting low-significance network connections and consequently under-fitting the most significant interactions. This is particularly relevant with respect to network structure identification, where the object is to distinguish significant, "direct" network connections from insignificant network interactions. In addition to the model observations, differences in the calculated sensitivity index distributions are also evident from experimental data analysis. Fig. S3 shows RS-HDMR with MR to be much more effective in characterizing network interactions as either strong or weak. Only one of the network interactions from individual data set analysis (See *Methods*) was calculated by RS-HDMR with MR to have a sensitivity index between zero and 0.15, and the range of observed sensitivity indices is from 1.02 (slight overfitting can result in $S_i > 1.0$) to zero. RS-HDMR without MR, however, shows a much flatter distribution of sensitivity indices, ranging from only 0.2 to

roughly zero.

2. Interpolative and Predictive Accuracy. In addition to biased sensitivity analysis, strong correlation amongst the input variables can lead to problems of overfitting and decreased interpolative and predictive accuracy. Over fitting also can arise under conditions of noisy and sparse sampling. As with network structure identification, these problems are only amplified when calculating higher-order RS-HDMR functions. Fig. S4 shows the difference in interpolative accuracy between second-order RS-HDMR analysis with and without MR, fitting data from the model with "Indirect Connection to x_9." Fig. S4 demonstrates that MR significantly increases RS-HDMR fitting accuracy. In this application, the residual variance from results using RS-HDMR decreases by over 95% upon implenting MR.

3 Multivariate Individual-Cell Data

Human primary naive $CD4^+$ T-cells were fixated after 15 minutes exposure to exogenous signaling cues and perturbation reagents, as described in Sachs *et al.* Flow cytometry was then used to simultaneously measure eleven different phospholipid and phosphorylated protein levels in individual cells [Akt (S473), Jnk (T183 and Y185), Raf (S259), mitogen-activated protein kinases (MAPKs) Erk1 and Erk2 (T202 and Y204), p38 MAPK (T180 and Y182), Mek1 and Mek2 (S217 and S221), protein kinase A (PKA) substrate phosphorylation, phospholipase Cγ (PLCγ Y783), PKC (S660), phosphatidylinositol 4,5-bisphosphate (PIP2), and phosphatidylinositol 3,4,5-triphosphate (PIP3)] .

Table S2 summarizes the employed perturbative conditions in the nine data sets used for this analysis. See Sachs *et al* for details regarding specific stimulating and inhibiting reagents used in the perturbation experiments, as well as protein and phosphorylation sites measured. Cytometry measurements from individual cells make up a total of 5400

eleven-dimensional data points from nine different data sets, with each set containing cell measurements observed under a unique perturbative condition. Two data sets are for general stimulatory conditions (d_1 and d_2). The remaining seven data sets are under protein-specific perturbative conditions.

Each of the nine data sets were analyzed individually. Thirteen pairs of data sets were also analyzed to examine the effect of the exogenous perturbations. Data sets d_3–d_9, with the exception of d_7, were paired with each of the two data sets (d_1 and d_2) describing the network under general stimulating conditions, for a total of twelve pairings. Data set d_7 was excluded because the perturbation does not inhibit any of the measured species to the same degree of specificity as the other perturbations. The pairing of data sets d_8 and d_4, where PKC is activated and inhibited, respectively, constitutes the thirteenth pairwise comparison data set.

Protein level data was transformed to a logarithmic scale and linearly normalized such that for a measured species, x_i, we have $0 \leq x_i \leq 1$ for all species in a given data set used for RS-HDMR analysis. RS-HDMR analysis was performed on 70% of the total data from each data set. The remaining 30% was used to test the accuracy of the predictive IO models. For each data set, multiple (10) subsets were used to iteratively analyze the data in order to validate the sensitivity analysis. Outlier data points with values falling greater than two standard deviations from the mean were excluded from analysis. The experimental data reported in Sachs *et al* was obtained online [4].

References

1. Wang S, Georgopoulos P, Li G, Rabitz H (2003) Random Sampling-High Dimensional Model Representation (RS-HDMR) with Nonuniformly Distributed Variables: Application to an Integrated Multimedia/Multipathway Exposure and Dose Model

for Trichloroethylene. J Phys Chem A 107: 4707–4716.

2. Li G, Rosenthal C, Rabitz H (2001) High dimensional model representations. J Phys Chem A 105: 7765–7777.

3. Chatterjee S, Price B (1977) Regression analysis by example. Wiley New York.

4. Sachs K, Perez O, Pe'er D, Lauffenburger D, Nolan G (2005) Causal Protein-Signaling Networks Derived from Multiparameter Single-Cell Data. Science 308: 523–529.
